# A proposed syntax for Minimotif Semantics, version 1

**DOI:** 10.1186/1471-2164-10-360

**Published:** 2009-08-05

**Authors:** Jay Vyas, Ronald J Nowling, Mark W Maciejewski, Sanguthevar Rajasekaran, Michael R Gryk, Martin R Schiller

**Affiliations:** 1 Department of Molecular, Microbial, and Structural Biology, University of Connecticut Health Center, 263 Farmington Ave. Farmington, CT 06030-3305 USA; 2 Department of Computer Science and Engineering, University of Connecticut, 371 Fairfield Rd., Storrs, CT 06269-2155 USA; 3 University of Nevada, Las Vegas, School of Life Sciences, 4505 Maryland Pkwy., Las Vegas, NV 89154-4004 USA

## Abstract

**Background:**

One of the most important developments in bioinformatics over the past few decades has been the observation that short linear peptide sequences (minimotifs) mediate many classes of cellular functions such as protein-protein interactions, molecular trafficking and post-translational modifications. As both the creators and curators of a database which catalogues minimotifs, Minimotif Miner, the authors have a unique perspective on the commonalities of the many functional roles of minimotifs. There is an obvious usefulness in standardizing functional annotations both in allowing for the facile exchange of data between various bioinformatics resources, as well as the internal clustering of sets of related data elements. With these two purposes in mind, the authors provide a proposed syntax for minimotif semantics primarily useful for functional annotation.

**Results:**

Herein, we present a structured syntax of minimotifs and their functional annotation. A syntax-based model of minimotif function with established minimotif sequence definitions was implemented using a relational database management system (RDBMS). To assess the usefulness of our standardized semantics, a series of database queries and stored procedures were used to classify SH3 domain binding minimotifs into 10 groups spanning 700 unique binding sequences.

**Conclusion:**

Our derived minimotif syntax is currently being used to normalize minimotif covalent chemistry and functional definitions within the MnM database. Analysis of SH3 binding minimotif data spanning many different studies within our database reveals unique attributes and frequencies which can be used to classify different types of binding minimotifs. Implementation of the syntax in the relational database enables the application of many different analysis protocols of minimotif data and is an important tool that will help to better understand specificity of minimotif-driven molecular interactions with proteins.

## Background

Minimotifs (also called Short Linear Motifs [SLIMs]), are short peptide sequences which play important roles in many cellular functions [1-3]. Many minimotif databases such as Minimotif Miner (MnM), Eukaryotic Linear Motif (ELM), phospho.ELM, DOMINO, MEROPS, PepCyber and HPRD have cataloged more than a thousand minimotif entries and are expected to have significant growth in the near future [1,4-10]. Each of these databases model functional minimotifs in some capacity, often using individualized annotation schemes useful for the subset of minimotif data being managed. As the amount of minimotif data continues to grow, there are several expected advantages to be gained from the use of a standardized syntax. A standardized syntax will facilitate exchange of data with different minimotif databases. Likewise, a standardized syntax will allow integration with other non-motif databases enabling researchers to examine the connection of minimotifs with new types of data (e.g. disease mutations, protein structures, cellular activities, etc.), providing new opportunities for data mining. A standardized syntax will also allow refinement of minimotif sequence definitions, reduce redundant data, and normalize future annotation efforts.

The authors have been the curators of the Minimotif Miner database for the past four years. In compiling and managing this large dataset, we have had a lengthy and detailed exposure to the functional annotations currently reported in the scientific literature. This unique perspective has afforded us the insight as to certain common features of the functional annotation of minimotifs. Here we propose a standardized definition for minimotifs that is currently being used within MnM and which can be broadly applied to all minimotifs including those in the aforementioned databases.

We have observed that all minimotif annotations are composed of two major categories, the covalent chemistry and the function of the peptide. The first component of a minimotif definition includes its sequence and modification information. Schemes for modeling the sequence of minimotifs are well established and have been adopted from previous work modeling protein domains[11,12]. The protein sequences of minimotif instances are sequence strings of amino acids represented using an alphabet of IUPAC single letter code amino acid abbreviations [13]. For example, the 'PKTPAK' sequence in Kalirin describes an instance or single occurrence of a minimotif. Higher level minimotif abstractions are often represented as consensus sequences or position specific scoring matrices (PSSMs). Consensus sequence definitions identify permissible positional degeneracy. PxxPxK is an example of consensus definition that describes multiple instances for proteins that bind to the SH3 domain of Crk; 'x' indicates that any of the 20 amino acids are allowed at the indicated position. Degeneracy can also be indicated for groups of amino acids that have similar chemical properties represented by a set of Greek symbols [14]. Consensus sequences can be represented as regular expressions in PROSITE syntax [12]. Probability-based PSSMs, like consensus sequences, represent the degeneracy at each position, but have the advantage that the probability of an amino acid at each position is explicit. PSSM are commonly represented as LOGO plots [15,16].

The sequence definitions described above, by themselves, have been found to be insufficient to describe many minimotifs which require additional covalent chemical modification. A set of rules for indicating post-translational modifications was previously defined by the Seefeld Convention [14]. One such rule is to indicate a phosphorylated residue by a lower case 'p' preceding an amino acid (e.g. RSxpSxP indicates the second Ser is phosphorylated in this 14-3-3 binding minimotif [17]). In our experience there are two important limitations imposed by the Seefeld Convention. First, the forced distinction between lowercase and uppercase character sets puts undesirable constraints on the implementation hardware/software; likewise the use of Greek characters to indicate degeneracy of amino acids with similar physical properties in minimotif definitions can also be problematic due to machine-specific character encoding. Second, this minimotif syntax is not extensible to all of the approximately 500 known posttranslational modifications, several of which have established roles in minimotif function [14,18]. For example, myristoylated residues and cis-proline bonds can not be enumerated using the Seefeld Convention. In this paper, we describe a model that overcomes these limitations for minimotif sequence definitions.

The second component of minimotifs is their biological function(s), which have generally been free-form descriptions in minimotif databases with no set standard. To our knowledge this minimotif subdomain of knowledge has not yet been modeled, which limits the ability to integrate data from different databases and hence their global usefulness. There are several ontologies that address domains related to minimotifs. The Gene Ontology (GO) defines a vocabulary for molecular and cellular functions and the association of these functions with gene products. While this ontology provides a useful resource for functional activities, the GO database is not designed to describe minimotif functions, nor capture important common attributes that are specific to minimotifs [19]. For example, the bind function in GO does not indicate the residues involved in an interaction, nor if any of these residues require any post-translational modifications. Likewise, the Protein Ontology, PSI-MOD, and RefSeq databases help to define entities that can be used for modeling minimotifs but are not sufficient by themselves for this purpose [20,21].

We provide a standardized semantic and syntactic definition of minimotifs gleaned from the data contained within MnM 2, and have executed its implementation by refactoring approximately 5000 minimotif annotations within MnM. As an example of the utility of this model and syntax, we demonstrate the use of the new database in classifying SH3 binding minimotifs.

## Results

### Minimotif Function Elements

A disambiguated and extensible semantic basis for minimotif functionality was derived from a set of rules which characterizes the approximately 5000 minimotifs in the Minimotif Miner (MnM) database [1] without information loss. We have not created a formal grammar, but rather a set of rules that characterize minimotif descriptions. For any minimotif clause, the syntax is *Minimotif *(subject), *Activity *(verb), and *Target *(object) which can be derived from a set of rules. We define these three major elements as follows:

*Minimotifs *consist of sequence definitions and sources. The sequence definition can be an instance, a consensus sequence, or a PSSM; all three classes of minimotifs are commonly reported in the literature. Instances represent primary data, whereas consensus sequences and PSSMs are interpretations of the data. *Minimotifs *may require one or more post-translation modifications such as phosphorylation or proline isomerization. In each motif, these modifications can be described by one or more residue names, type(s) of modification, and position(s) in the *Minimotif *sequence. Another approach for modeling residue modifications could be the atomic model previously described [22]. A source is the protein or peptide that contains the minimotif sequence. For example, in ' [PKTPAK in Kalirin] [binds] [Crk]', 'PKTPAK' is a sequence definition and 'Kalirin' is the minimotif source [23]. Alternatively, PxxPxK is a consensus definition that describes a consensus sequence for multiple instances.

*Targets *are proteins, nucleic acids, carbohydrates, lipids, small molecules, elements, metals, drugs, or complexes. In the case of proteins and nucleic acids, *Targets *may be associated with sequence definitions. *Target *proteins may contain domains as defined by the Conserved Domain Database [24], belong to a hierarchical classification based on fold [25] or refer to determined structure elements [26]. In the above example of the PKTPAK minimotif, the *Target *'Crk' can be expanded to be more specific '1st SH3 domain of Crk'; referring to the N-terminal of two SH3 domains in Crk.

*Activities *are the actions of minimotifs and all minimotif activities can be generally classified as binds, modifies or traffics. The 'Binds' *Activity *describes an interaction of a protein containing a minimotif with another molecule. The 'Modifies' *Activity *defines a chemical change to a minimotif sequence that can be further subcategorized into enzymatic activities such as phosphorylates, amidates, geranyl gernaylates, cleaves etc The 'Traffics' *Activity *describes minimotif sequences required for a protein to be shuttled between cell compartments or other specific locations within or outside of cells.

In a number of minimotifs, a *Minimotif *and *Activity *are known, but the *Target *has not yet been identified or it is not yet known if the interaction of the *Minimotif *with the *Target *is direct. This information is still useful, thus we utilize a 'Required' *Activity *category which indicates that a minimotif sequence is necessary for a molecular or cellular activity. For example, the PNAY minimotif in Crk is required for Abl kinase activation [27]. In this case, Abl kinase activation is a subcategory of 'Required'. As in this example, the *Target *is null for the 'Required' *Activity*.

### Minimotif Syntax

In order to combine these major minimotif elements and the minimotif sequence definition into human-interpretable semantic sentences we have defined 22 different attributes of minimotifs (Table 1) and derived the set of syntax rules listed below. Our goal was to identify a minimal set of rules that combine minimotif elements in order to regenerate valid minimotif sentences for the ~5000 minimotifs in the Minimotif Miner database. Valid minimotif sentences are based on these syntax rules, and biological entity categories of innumerable size (i.e. protein domains, protein names, molecule names, etc.).

**Table 1:** Attributes of a minimotif definition

#	**Attribute**^1^	Valid values and description
1	Motif sequence type	(Consensus, instance, PSSM) type of sequence definition

2	Motif sequence	Any consensus, instance, or PSSM describing a minimotif protein sequence

3	Required modification	description of chemical change to minimotif sequence

4	Motif source name	The name of protein or peptide that contains the minimotif

5	Motif source accession number	Swiss-Prot, RefSeq accession numbers for protein sequences containing the minimotif

6	Motif start position	Integer start position of the minimotif in motif source accession number

7	Motif source type	(Peptide and/or protein) indicates whether minimotif was investigated as a peptide fragment or in a protein domain

8	Activity	(binds, modifies, requires, traffics) the action of the minimotif

9	Subactivity	A more detailed description

10	Activity modification	Description of activity that covalently changes a minimotif sequence

11	Target name	The name of the molecule that acts upon the minimotif

12	Target accession number	If the target is a protein, the Swiss-Prot or RefSeq accession number(s) for Target protein sequence(s). The target can be a complex

13	Target type	(Peptide and/or protein) indicates whether *Target *was investigated as a peptide fragment or in a protein domain

14	Target domain	(any domain in the CDD) protein domain in the minimotif *Target*

15	Target domain position	Integer that indicates the relative location of a domain relative to its N-terminus for proteins that have more than one copy of the same domain

16	Target site	Integer for site where a minimotif binds a molecule, if more than one site is known

17	Subcellular localization	Region of the cell where the minimotif activity occurs

18	Affinity	(K_d_, IC50, K_m_) measurement of affinity of minimotif for its target

19	Structure	(PDB accession number) for a structure of the minimotif in complex with its target. A related attribute is 'related structures' of the minimotif source or target.

20	Experimental evidence	(X-ray, NMR, Phage display, peptide mapping, alanine scanning mutagenesis, evolutionary conservation, mutagenesis, modeling, deletion mapping, peptide binding, peptide competition, full-length protein, Surface Plasmon Resonance, ITC, SPOT array, Far-western, Co-immunoprecipitation, yeast 2-hyrbid, pulldown) different types of experimental evidence that supports a minimotif sentence.

21	Minimotif reference	(PubMed identifier or PDB accession number) indicates the references source(s) of the data supporting the minimotif definition

22	Database reference	Cross reference ID to other database that contains similar minimotif definition.

^1 ^1 Attributes are broken up into 4 sections related to the *Minimotif *(1-6), *Activity *(7-9), *Target *(10-15), and properties (16-19) of *Motif*/*Activity*/*Target *minimotif sentences.

#### Syntax Rules

Format: Minimotif elements in quotes are variable and defined in Table 1. Additional definitions are shown in Table 2. Bold text does not change and italicized elements are optional. Each minimotif function conforms to one of four rules (binds, modified, traffics, required).

**Table 2:** Definitions of minimotif elements

Element	Definition
Minimotif	The covalent chemistry of a peptide segment represented by a sequence definition and any required modification and minimotif source

Minimotif sequence	An instance, consensus sequence, or PSSM that describes a peptide minimotif of less than 15 contiguous residues

Required modification	A change in the covalent chemistry of a minimotif sequence

Motif Source	The protein or peptide that contains the motif

Target	The molecule related to a minimotif by an activity

Activity	The action of the minimotif

Binds	Type of activity that involves a direct interaction between two or more molecule species

Modifies	Type of activity where the minimotif has a change in its covalent chemistry

Traffics	Type of activity where a protein moves between cellular compartments

Required	Type of activity where a minimotif is required for a chemical or cellular process

Chemical process	An event that results in a change of covalent bonds on a molecule

Cellular compartment	A place in the cell that can be discerned by the localization of at least one molecule

Peptide	Short polymer of amino acids

Protein	Polymer of amino acids

Domain	A region of a protein that folds independently.

Domain position	Location of a domain type in a protein that has more than one copy of a domain type relative to the N-terminus

Cellular process	An event or series of events that results in an observable change in a cell

'Minimotif' = 'Minimotif Sequence' ('*Required Modification*') **in **'Peptide' OR 'Protein'

'Protein target' = '*Domain position*' '*domain' ****domain of***' 'Protein'

'Target' = 'Molecule' OR 'Protein target'

'Required modification' = '*Amino acid*' '*Position*' **residue is **'*posttranslational modification*'

'Activity modification' = '*Amino acid*' '*Position*' **residue is **'*posttranslational modification*'

*BIND RULE: '*Minimotif' **binds **'Target'

*MODIFICATION RULE*: 'Minimotif' **is modified by the **'enzyme activity' **of the **'Protein target' *('activity modification')*.

*TRAFFIC RULE: '*Minimotif' **is trafficked by **'Target' **to **'Cellular compartment' OR 'Minimotif' **is trafficked to **'Cellular compartment'

*REQUIRED RULE: '*Minimotif' **is required for '***Chemical Process*' OR '*Cellular Process*'

#### Syntax Examples

*BIND RULE*: [IL]xxxxNPxY (tyrosine 497 residue is phosphorylated) in Interleukin 4 receptor **binds **PTB domain of IRS-1 [28].

*MODIFICATION RULE*: GRG in myelin basic protein **is modified by the **N arginine methylation activity of PRMT1 (Arginine 107 is methylated) [29].

*TRAFFIC RULE*: WHTL in Synaptotagmin **is trafficked to **synaptic vesicles [30].

*REQUIRED RULE*: GKFC in peptide **is required for **cell adhesion [31].

### Minimotif Model and Implementation

**Figure 1:**
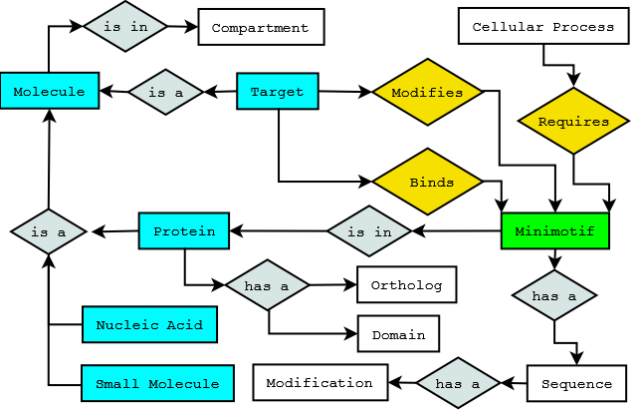
**Entity-relationship diagram of a conceptual minimotif data model**. *Activities *are colored orange; relationships are gray; molecules are cyan. There are properties of a *Motif/Activity/Target *in the database that are not present in this conceptual diagram.

The minimotif syntax was abstracted as a conceptual data model, which was used to derive logical and physical data models. An entity-relationship (ER) diagram of our conceptual data model is shown in Figure 1. The primary objects in the ER diagram are the *Minimotif *(green), *Activities *(orange), and *Target *(Cyan), each of which contains details regarding their attributes. Each *Minimotif *has a sequence and may have a modification (e.g. tyrosine phosphorylation in BIND RULE). All *Minimotifs *are in proteins which may have orthologues and domains. Each *Minimotif *can have a *Target *which is a molecule (Protein, Nucleic acid and small molecule are molecules; cyan). Molecules are in cell compartments. The *Target *has two relationships with the *Minimotif *(orange): modifies refers to a change in chemistry of the *Minimotif*, thus the *Target *is an enzyme in this case (MODIFIES RULE). For example, a *Minimotif *that is cut by a protease is chemically modified by an enzyme. The *Target *can also bind the *Minimotif *(BIND RULE). In the case where a *Target *molecule is not known, the *Minimotif *may be required for some *Activity *as in the REQUIRED RULE above. The TRAFFIC RULE is not represented in this diagram, but a *Minimotif *is trafficked by a *Target *from one cell compartment to another; the *Target *need not be known for the TRAFFIC RULE.

**Figure 2:**
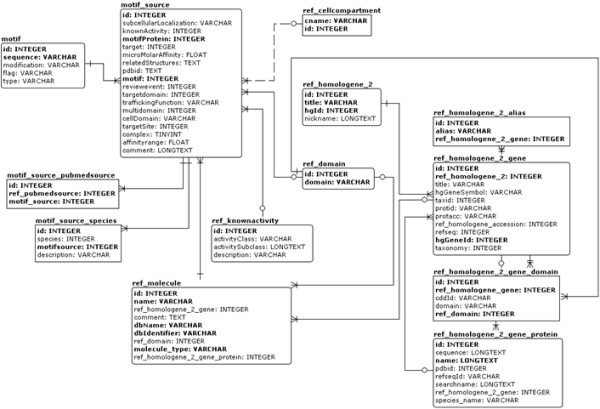
**A physical implementation of the conceptual minimotif data model in MySQL**. Relationships between tables are indicated. Three convergent lines pointing outward from a table indicate its dependency on another table. A circle or bar at the end of a line indicates that a relationship is optional or mandatory, respectively.

The physical implementation of the database is shown in Figure 2. The design of the minimotif relational database shows an intersection table (*motif_source*) of the *Minimotif, Activity*, and *Target *tables. Each minimotif in the database table has its own specific attributes such as minimotif type (consensus sequence or instance), a structure from the Protein Data Bank, an affinity for the *Minimotif/Target *complex, and published experimental techniques that support the *Minimotif/Activity/Target *relationship.

We have previously reported the MnM 2 database which contains more than 5000 minimotifs [2]. We have now refactored the MnM 2 database to use controlled vocabularies. These include the Gene Ontology (GO; the *Activity *term names and id's for common molecular functions), NCBI Taxonomy for id's and species names, NCBI Conserved Domain Database (CDD; the names and identifiers for protein domains in motif *Targets*), NCBI Reference Sequences (RefSeq; for *Target *and *Minimotif *source protein names and ids), Human Proteome Organization (HUPO; for experimental evidence names and id's), Psi-Mod for post translational modifications of *Minimotifs*, and the Protein databank (PDB, for accession numbers for protein structure files). The new relational database that uses these controlled vocabularies enforces, normalizes, integrates, and explicitly defines the minimotif semantics. Details concerning the database are in **Methods**.

The minimotifs in the Minimotif Miner (MnM) database were refactored and implemented in MnM 2 [2]. Our implementation of this model supports an integrative, semantically-rich minimotif analysis via the Structured Query Language (SQL), and importantly, is compatible with external motif analysis algorithms. This implementation enables extraction of groups of *Minimotifs *which share common values for any subset or combinations of subsets for the 22 different attributes in the model (Table 1). A set of 10 rules can be used to regenerate structured unambiguous human readable annotations [see Additional file 1].

We have built a user interface that enables users to query this database. This webpage is available as a link from the MnM 2 website. Users can select identifiers or text based descriptions from controlled vocabularies to query the database. For example, all SH3 binding motifs can by identified by selecting this domain from the CDD controlled vocabulary for domains [24]. Many minimotif attributes can be queried from this page.

Once the query system is used to retrieve and group primary minimotif data (instances), interpretations of this data are often the next step in minimotif analysis. The interpretations of this data most commonly reported in the literature are consensus sequences, PSSMs, and groupings of families of minimotifs; these can be automatically generated based on query results generated by the aforementioned query system.

Often a single laboratory does an experiment that identifies a consensus sequence, PSSM or grouping. MnM stores individual instances as reported in the literature, as well as inferred consensus sequences as reported by the authors. Our new query page has the advantage that consensus sequences, PSSMs or families of motifs can be generated from user-selected instances from one or more independent studies. Thus, this tool can be used to study groupings, consensus sequences, and PSSMs, which can vary significantly between different studies. Once groupings of instances are selected from the new query page, users can then generate consensus sequences or PSSMs.

### Grouping SH3 Domain Binding Minimotifs

There are many advantages expected to be gained by the use of a standardized minimotif syntax and query system. One such advantage is the simplified clustering of data within the database based on these new syntactical rules. As a case example, we classified 1363 SH3 binding minimotifs queried from the MnM 2 database. We selected this collection of data because of both the large number of reported SH3 binding minimotifs and the growing number of reported consensus sequences (e.g. PxxP, RxxPxxP, and PxxPxx [KR]). We posed a number of questions which would have been difficult to address without the syntax, but which are now easily addressed by querying the new relational database: Which SH3 consensus sequences are most common? How many SH3 binding consensuses are present in different instances? Do SH3 minimotifs bind to the same site? Is there a residue preference for degenerate positions?

A number of these questions had already been answered in an *ad hoc *fashion, but our goal in this case study was to address these questions in a systematic manner. Additional details for this analysis are provided [see Additional file 1].

**Figure 3:**
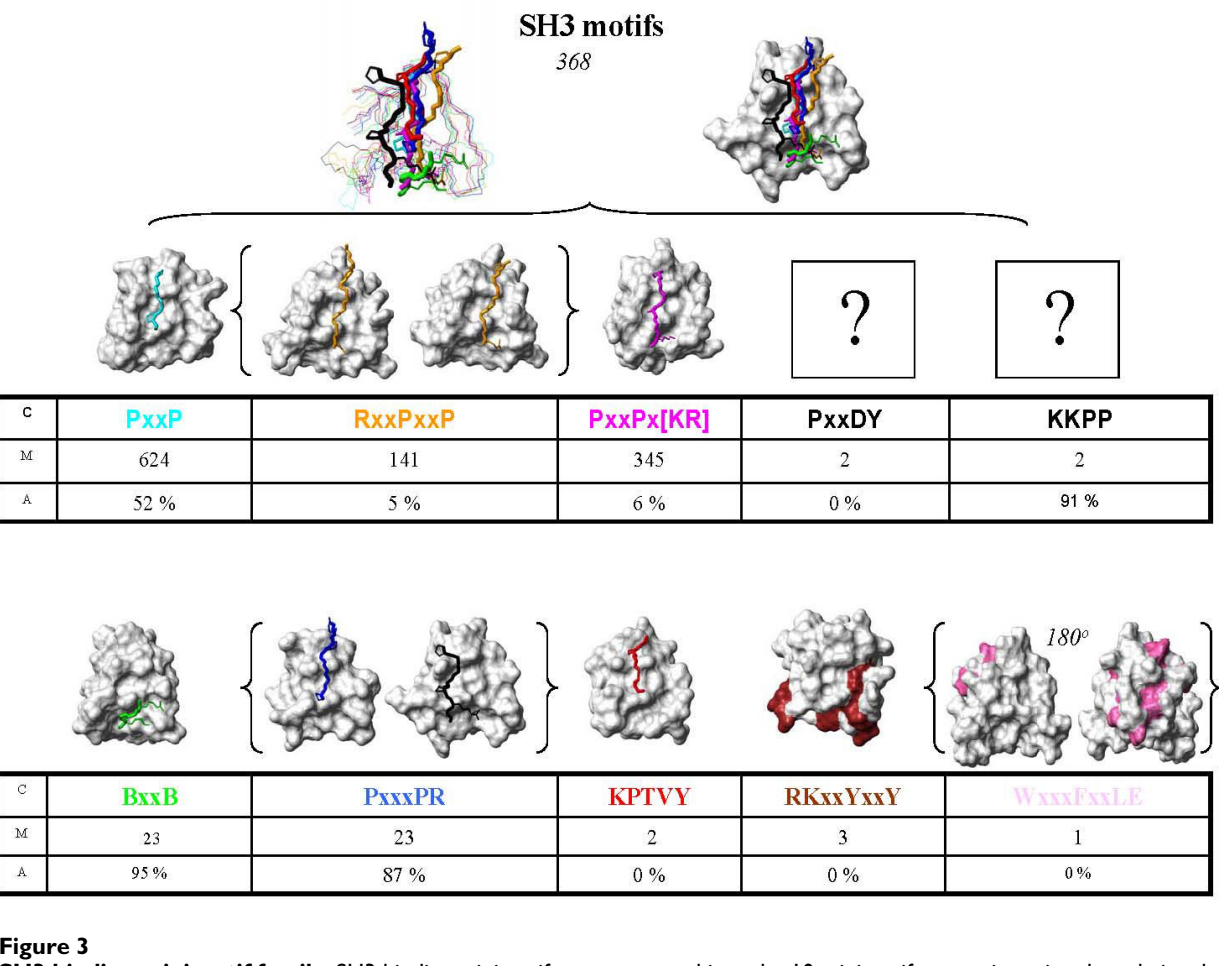
**SH3 binding minimotif family**. SH3 binding minimotifs were grouped into the 10 minimotif categories using the relational database and Shannon Information Content similarity metric. Surface plots of structures identified for 8 of the 10 group (1ZSG, black; 1NM7, pink; 1AZE, cyan; 2BZ8, blue; 1CKA, magenta; 1OPL, red; 1RLQ orange; 1H3H, green; 1NYG, brown) are shown. The carbon backbones of SH3 domains were fit using Molmol with residues in the β1 and β4 sheets, and the 3-10 helix, to an RMSD of 0.9 [33]. An overlay of each SH3 domain carbon backbone with its peptide minimotif is color matched and relevant minimotif side chain bonds are represented as thickened lines; the surface plot for the overlay is derived from the 1ZSG structure). Structures of the ligands for the RKxxYxxY and WxxxFxxLE minimotifs are not known, but the binding sites on the SH3 domains derived from NMR chemical shift mapping experiments are indicated. RxxPxxP and PxxxPR minimotifs show structures with the peptides in opposing orientations. The consensus sequences (C), total number of minimotifs for C (M), and percentage of potentially ambiguous ligand instances (A) in the MnM 2 database are indicated.

The groups of SH3 binders were extracted by custom SQL statements filtering *Minimotifs *by type (consensus vs. instance), *Target *(SH3 containing proteins), and *Activity *(binds). This resulted in 1363 (741 unique) SH3 binding minimotifs, which could further be segregated into 69 consensus sequences and 672 instances. These sequences were compared inside our database for similarity based on the Shannon Information Content similarity metric as implemented by the Comparimotif library [32]. This analysis resulted in 10 minimotif groups that describe all SH3 binding minimotifs in the database (Figure 3). Details concerning the clustering analysis, queries, and results that lead to the distinct minimotif groups are provided [see Additional file 1].

### Structural analysis of SH3 ligands

In order to better understand how these 10 SH3 binding minimotif groups were related to each other, we analyzed their known SH3/ligand complex structures. We queried the Minimotif Miner database and located representative structures for eight of the 10 groups. The *fit *function of Molmol was used to align the backbones of the eight SH3 domains using 6 residues in the β1 sheet, 4 residues in the 3-10 helix and 6 residues in the β4 sheet [33]. The root mean squared deviation (RMSD) for alignment of the backbone residues in these regions was 0.9 Å indicating a good alignment (Figure 2). We then examined the relationships of the binding sites of the different minimotifs by adding the sidechain bonds of the conserved residue positions and backbone atoms for each minimotif. For two structures we were only able to identify the binding sites based on nuclear magnetic resonance chemical shift mapping experiments [34,35].

Our analysis revealed that although SH3 domains are most commonly discussed for their ability to bind PxxP containing peptides, members of the SH3 domain family bind several different consensus sequences and have specialized structural interfaces. Of the 10 minimotif groups, many used different binding pockets on the SH3 domain. Four minimotifs bound in a similar region to the standard PxxP binding site (RxxPxxP, BxxB, PxxxPR, and KPTVY). The BxxB (B = basic) shares only one of two binding pockets with PxxP as previously noted [36,37]. Two of the motifs (RxxPxxP and PxxxPR) were found to bind in two different orientations with the peptides flipped ~180° in the binding sites. Two other consensus sequences bound previously identified alternative sites not near the PxxP site, and two had no structural information. This analysis confirms the distinction of the minimotif clusters derived by the sequence based-analysis.

### Most SH3 domain binding peptides have multiple consensus sequences

Until recently, BxxB, PxxxPR, and several other types of SH3 binding minimotifs were not known. Given that there were 10 different types of SH3 binding consensus minimotifs, we wanted to know to what extent did previously studied ligands have multiple consensus sequences. We designed a query (query 9) that assessed how many consensus sequences were present in each ligand excluding the pairing of PxxP with RxxPxxP and PxxPx [KR] because these minimotifs are children of PxxP.

The average number of minimotif consensa per SH3 ligand was 2.3 indicating a tendency for each ligand sequence to have multiple SH3 consensus sequences. In the most extreme examples the SPTPPPVPRRGTHT, QPPVPSLPPRNIKP, KKPPPPVPKKPAKS, RRPPVPPR, and RRAPPPVPKKPAKG ligands each have five of the 10 different SH3 binding consensus sequences. For each consensus sequence, we have also reported the percent ambiguity in Figure 3 which is the percentage of each minimotif for which there are multiple consensus sequences. It is obvious from this analysis that a high proportion of previous SH3 binding experiments assessed ligands with potential to have multiple ligand binding modes. Thus, the majority of SH3 binding data may be subject to ambiguous interpretation (Figure 3). In interpreting many previous SH3 binding experiments, new ligand binding modes may now need to be considered in the experimental interpretation. Our database contains only 50 of the 270 known human proteins with SH3 domains, thus the 10 SH3 minimotif groups we identified may become even more complex with a comprehensive analysis of all SH3 domains.

### All SH3 domain binding peptides have basic residues

To further characterize the SH3 binding landscape, we performed analysis of residue content in all SH3 ligands using queries as described in methods. Compositional analysis showed a high preference for proline (4.2 fold), arginine (1.7 fold), and lysine (1.8 fold)(Table 3). In fact, all SH3 ligands in the database contained either a lysine or arginine, suggesting that a positive charge may be an important factor in ligand binding to SH3 domains. Another study has previously suggested a role for positively charged residues in SH3 domain interactions [38]. Consistent with this observation, the least enriched residues in SH3 ligands were the negatively charged residues.

**Table 3:** Residue frequencies in SH3 domain ligands

Residue	Total Count	Composition (%)	Enrichment (fold)
A	554	7.4	1.0
C	118	1.6	0.7
D	102	1.4	0.3
E	100	1.3	0.2
F	206	2.8	0.8
G	275	3.7	0.6
H	54	0.7	0.3
I	171	2.3	0.6
K	764	10.2	1.8
L	697	9.3	1.0
M	64	0.9	0.4
N	150	2.0	0.6
P	2026	27.2	4.2
Q	200	2.7	0.6
R	752	10.1	1.7
S	404	5.4	0.7
T	310	4.2	0.8
V	336	4.5	0.8
W	59	0.8	0.7
Y	102	1.4	0.5

The overall average calculated charge of SH3-binding peptides in our database was +3.2 ± 1.4 (average length of 12.1 ± 3.1 residues); this calculation is based on summing charges of basic and acid residues assuming a neutral pH. Of nine other groups of minimotifs with common domain targets in MnM 2 only minimotifs for Calmodulin (n = 31) and 14-3-3 (n = 44) had net positive charges of 3.0 ± 1.3 and 1.0 ± 0.9, respectively; PDZ (n = 1089), SH2 (n = 952), kinase (n = 206), PTB (n = 168), protease (n = 93), FHA (n = 67), WW (n = 27) and phosphatase (n = 25) domains had ligands or substrates with an average neutral or net negative charge.

Collectively, these query results strongly suggest that known SH3 peptide ligands have a more positive overall charge than proteins in the human proteome. It is important to note that when restricting the SH3 ligand query to non-BxxB sequences, the average ligand charge was still +2.2 ± 1.2. Only 11 of the 1363 sequences had a neutral or negative charge and several of these were for WxxxFxxLE and PxxDY minimotifs, which have few instances in the dataset.

## Discussion

We have developed a syntax with a set of rules that describes the more than 5000 minimotifs in the MnM database. While this syntax is complete for the data currently managed by MnM, we will actively continue to develop and expand this model to support additional types of data. The syntax is important because it enables the use of controlled vocabularies through defined rules, integration with other types of databases, exchange of data between minimotif databases, and the ability to address difficult questions that are facilitated through mining of minimotif data.

Current approaches for defining the covalent chemistry of minimotifs are not without limitations, beyond the post-translational modifications discussed earlier. The most commonly used representation of a motif is a consensus sequence. The definition of the word consensus does not necessitate that all members of a group conform, thus consensus sequences, while having the advantage that they can be used to group a number of instances, can also introduce ambiguity. For example, Calmodulin binding minimotifs have several members that do not conform to consensus sequences [39].

We have decided not to model a relationship between instances and their consensus sequences because these can be reconstructed through database queries that use a wider set of data. However, this approach remains to be tested with rigor and consensus sequences with nonconforming members may prove difficult. There are likely to be other ways that consensus sequences are limiting, for example, our SH3 minimotif analysis suggests that this binding minimotif should have an overall positive charge, which can not be represented by a consensus sequence. Furthermore, our semantics currently rely on consensus sequence definitions and our syntax does not support PSSMs. While a thorough discussion of sequence definition limitations is beyond the scope of this paper, we expect that through continued annotation using our standardized syntax we will able to identify all anomalies in our model and adjust it accordingly.

Through our work on minimotifs, we recognized a number of other important limitations that will need to be addressed in the future. Several attributes of minimotifs could be modelled better. For example, some *Targets *of motifs are complexes, rather than single proteins. Furthermore, a specific structural conformation of a protein may be specific to a *Minimotif *or *Target*. Wherever possible we have tried to use controlled vocabularies, but a number of attributes could expand on this theme. We could better use vocabularies for activities and subcellular localizations from the GO database. However, we have recognized that all minimotif, and perhaps molecular activities, fit into the general categories of binds, modifies, or traffics, a basic grouping of function not implemented in GO. Alias names of proteins also present a problem with redundancies, but this is a problem endemic to many biological databases. While many previous minimotif descriptions in the literature use elements of the syntax we propose, the syntax is not always structured the same way, making automated annotation or restructuring of previous literature difficult. Finally, there is no guarantee that all future minimotif functions we identify will fit in our model.

We have shown that implementation of the syntax is useful. Our analysis of SH3 binding minimotifs identified over 1000 minimotifs that cluster into 10 major groups. The majority of these groups bound to a similar site but, the specific contacts in the interaction were generally not conserved between groups. Thus, it seems that while the evolutionary pressure for binding to the SH3 domain is strong, the precise mechanism of binding can vary. This SH3 minimotif analysis emphasizes the necessity of standardizing minimotif semantics and sequences in a well-modeled database with a query system that can be used to manage data from a collection of related studies. The data-driven classification provides a solution to grouping minimotifs based on a broad collection of experiments with reduced bias towards any individual peptide screen or study. The semantics and relational database are important in this process because a large amount of data can be normalized and because sequence similarity is not the only indicator of functional similarity. For example, PLPP and SKSKDRYY possess similar activities even though they do not share a single residue in common [40,41].

## Conclusion

Information inconsistency arising from informal semantics is always a limitation for data integration. The minimotif semantics described here, along with the data model and its implementation, enable the computation of functional equivalence between minimotifs. This linguistic scheme is similar to one recently suggested by Gimona [42].

The syntax will facilitate many types of computational analyses of minimotifs. We are now able to generate specific subsets of data based on any of the 22 attributes of minimotifs. For example, the database facilitates refining sequence definitions similar to the recent refinement of a sumoylation minimotif [43]. The normalized syntax will allow exchange of data with other databases, reduce redundancies, and provides a framework for future annotations. The syntax also facilitates minimotif classification, as done for SH3 domain binding minimotifs in this paper.

## Methods

### Database Design

Our theoretical model of minimotif semantics is only useful if it is logically understood by a machine, thus the reason why we built a relational database. It is typical to implement database relationships in ways which exceed the complexity of the theoretical data model on which they are based (for performance and practicality reasons). Because many *Targets *can also be *Minimotif *containing proteins, and the three *Minimotif*/*Activity/Target *components are only related by experimental work, many additional tables were needed to link information for these components.

Full database documentation is provided [see Additional file 2]. Since the most important elements of our database are those which directly model the semantics, a mapping between our conceptual model and its physical implementation is provided in a table in Additional file 1. The physical model also includes many other federated data sources which are not in the conceptual model such as the gene alias names (ref_homologene_2_gene_alias), and minimotif annotation literature sources (motif_source_pubmedsource) which are linked to the ref_pubmedsource table (not shown). More information regarding these relationships is in Additional file 2.

Additional tables in the database were used for data mining. For example, Motif_source_motif_group groups minimotif_source records and ref_amino_acid is a table of all amino acids. The motif table contains the minimotif amino acid sequence and any post-translational modification to the sequence. Each minimotif is associated with one motif_source record, which is an intersection point for two ref_molecule records (one being the minimotif containing protein, and one being the molecule type of the target which the minimotif acts upon). The target is optional depending on the annotation rule.

Each ref_molecule entry can be optionally associated with either a RefSeq protein and/or a HomoloGene cluster, and additionally may have a ref_domain record (which is a federation of the NCBI Conserved Domain Database (CDD)) [24]. These clusters are important because many minimotif functions are conserved across species boundaries, allowing us to group RefSeq proteins which serve as minimotif targets.

**Clustering of SH3 minimotifs [see Additional file **1**]**

## Authors' contributions

JV, RJN, MRS, MRG, and SR developed the minimotif semantic and syntax. JV, MRS, MRG, and MWM contributed to the design of the minimotif data model. JV implemented the data model in a MySQL database. Refactoring and annotation of minimotifs into the minimotif data model was carried out by MRS. JV and MRS conducted the analysis of SH3 binding minimotifs. MRS, MRG, and JV prepared the manuscript and all authors were involved in editing. All authors read and approved the final version of the manuscript.

## Supplementary Material

Additional file 1 **Supplementary Methods, Data, and Results**. Supplementary methods and results for database design and clustering SH3 binding minimotifs. Click here for file

Additional file 2 **Database Documentation files**. File of documentation of the MySQL data model. Click here for file
