# Supplementary material for: A proposed syntax for Minimotif Semantics, version 1
Source: BMC Genomics. 2009 Aug 5;10:360. doi: 10.1186/1471-2164-10-360 (PMC2733157; doi:10.1186/1471-2164-10-360)
Supplement: Additional file 1 — Supplementary Methods, Data, and Results. Supplementary methods and results for database design and clustering SH3 binding minimotifs. [file 1471-2164-10-360-S1.doc]

## Supplementary Data.

## Supplementary Table 1. Physical to conceptual data model mapping.

| **Physical Model** | **Conceptual Model** | **Purpose** |
| --- | --- | --- |
| Motif | Motif | Defines the motif sequence, and any post‑translational modification |
| ref_knownactivity,motif_source | Activity | Defines the motifs activity for a given annotated motif. |
| ref_molecule, motif_source | Target | Defines the biological target of an annotated motif. |
| ref_homologene_2_gene_protein | RefSeq | Defines the RefSeq record for a motif containing protein or its target. |
| ref_homologene_2 | HomoloGene | Defines a HomoloGene cluster for any protein. |
| ref_domain | CDD | Defines types of protein domains |

### Supplementary Table 2. Rules used to regenerate annotations from database tables.

| **Rule #** | **Condition** | **Rule** |
| --- | --- | --- |
| 1 | ([Activity] = binds) AND ([Required Modification] = Instance) AND (Target Name domain = empty or null) AND ([Required Modification] does not = none) | [Motif Sequence] in [Motif source name] [Activity] [Target Name]; [Required Modification] |
| 2 | ([Activity] = binds) AND ([Required Modification] = Instance) AND (Target Name domain = empty or null) AND ([Required Modification] = none) | [Motif Sequence] in [Motif source name] [Activity] [Target Name] |
| 3 | ([Activity] = binds) AND ([Required Modification] = Instance) AND (Target Name domain = is not empty or null) AND ([Target domain position] = empty or null) | [Motif Sequence] in [Motif source name] [Activity] the Target Name domain Target Name domain of [Target Name]; [Required Modification] |
| 4 | ([Activity] = binds) AND ([Required Modification] = Instance) AND (Target Name domain = is not empty or null) AND ([Target domain position] = is not empty or null) | [Motif Sequence] in [Motif source name] [Activity] the [Target domain position] Target Name domain Target Name domain of [Target Name]; [Required Modification] |
| 5 | ([Activity] = binds) AND ([Subactivity] contains trafficked) AND ([Required Modification] = Instance) AND ([Required Modification] = none) | [Motif Sequence] in [Motif source name] binds [Target Name] and is [Subactivity] [Subcellular Localization] |
| 6 | ([Activity] = binds) AND ([Subactivity] contains trafficked) AND ([Required Modification] = Instance) AND ([Required Modification] is not = none) | [Motif Sequence] in [Motif source name] binds [Target Name] and is [Subactivity] [Subcellular Localization]; [Required Modification] |
| 7 | ([Activity] = requires) AND ([Required Modification] = Instance) AND ([Required Modification] = none) | [Motif Sequence] [Subactivity] requires [Required Modification] motif in [Motif source name]; Target Name is [Target Name] |
| 8 | ([Activity] = requires) AND ([Required Modification] = Instance) AND ([Required Modification] does not = none) | [Motif Sequence] [Subactivity] requires [Required Modification] motif in [Motif source name]; Target Name is [Target Name]; [Required Modification] |
| 9 | ([Activity] = modifies) AND ([Required Modification] = Instance) AND ([Required Modification] = none) | [Motif Sequence] in [Motif source name] is [Subactivity] by [Target Name]; [Activity Modification] |
| 10 | ([Activity] = binds) AND ([Required Modification] = Instance) AND ([Required Modification] does not = none) | [Motif Sequence] in [Motif source name] is [Subactivity] by [Target Name]; [Activity Modification]; [Required Modification] |

**Supplementary Table 2** defines the rules for generating human readable annotations from the structured attributes of the minimotif syntax. The syntactical attributes can be acquired by joining tables in the database. The value of different attributes in each condition for a minimotif determines which rule is used.

**Supplementary Results**

SH3 binding motif clustering. In order to determine SH3 domain binding motifs, a query against the ref_knownactivity, ref_molecule, motif_source, motif and ref_domain tables was executed to join their data (*query 1)*. The resultant cluster of motif *sequences* from this data set consisted of 741 distinct sequences (69 consensus sequences and 672 instances), with 59 *Target* (SH3 containing) proteins and 372 source (*Motif* containing) proteins. At this point, we have utilized our semantic model of minimotif function to derive a data set resulting from a very specific linguistic analysis which can now be analyzed for minimotif groupings. Several database procedures were needed for this analysis (queries 1-9).

## Supplementary Table 3. Queries for SH3 binding minimotif analysis.

| **Query Number** | **Syntax** |
| --- | --- |
| 1 | *Select sequence, '#', ref_molecule.name from motif, motif_source, ref_knownactivity, ref_molecule, ref_domain where motif_source.motif=motif.id, ref_molecule.id=motif_source.target, and ref_molecule.refDomain = ref_domain.id and ref_domain.domain = ‘SH3’ and ref_knownactivity.Activity=’binds’ and motif.type IS NOT 'Consensus'* |
| 2 | *Select motifClass,count(*),(select count(*) from motif_comparison), avg(score) from motif_comparison where score > 1 group by motifClass order by count(*)* |
| 3 | *Select sh3_group.rxp, count(0)/(select count(*) from lexica)*  *from sh3_group join lexica where lexica.sequence regexp (sh3_group.rxp) group by sh2_group.rxp union select 'NOT PXXP', count(0), count(0)/(select count(*) from lexica) from lexica where not isPxxP(lexica.sequence)* |
| 4 | *declare totalresidues int;select sum(length(m.sequence)) into totalresidues from sh3_binding_motifs_sandbox m; select a.letter,sum(substrCount(s.sequence,a.letter)) rawTotalCount, 100*sum(substrCount(s.sequence,a.letter))/totalresidues as percentComposition, 100*sum(substrCount(s.sequence,a.letter)>0)/totalresidues as rawAmountContaining, (100*sum(substrCount(s.sequence,a.letter))/totalresidues)/enric.percent as percentCompositionNormalizedToProteome from sh3_binding_motifs_sandbox s, ref_amino_acid a, ref_aa_enrichment_human_proteome enrich where enric.aa=a.`letter`group by a.letter* |
| 5 | ***q****Select motifClass,count(*),(select count(*) from motif_comparison), avg(score) from motif_comparison where score > 1 group by motifClass order by count(*)* |
| 6 | ***number of SH3 containing proteins in human proteome:*** *‘Select distinct ref_homologene_2 from ref_homologene_2 h, ref_homologene_2_gene_domain d,ref_homologene_2_gene g where domain =<domain> and d.ref_homologene_gene=g.id and g.ref_homologene_2=h.id’* |
| 7 | ***number of unique SH3 binding sequences:*** *‘Select distinct sequence  from motif,motif_source,ref_molecule,ref_knownactivity a where a.Activity ='binds'  and motif_source.knownActivity=a.id and motif_source.motif=motif.id and ref_molecule.id=motif_source.target and ref_molecule.ref_domain=(select id from ref_domain where domain='SH3') order by sequence and not sequence regexp('x') and not sequence like '%[%'‘* |
| 8 | ***charged character of SH3 binding sequences*** *: ‘Select avg(getPeptideCharge(s.sequence)) from human_proteome as s UNION*  *select avg(getPeptideCharge(s.sequence)) from distinct sh3_binding_lexica_type group by s.sequence regexp (‘[KR]..[KR]’)’* |
| 9 | *‘Select avg(s.cnt) from (select count(*) as cnt from motif_source_motif_group where group_title='SH3' and motif regexp(group_rxp) group by motif) s’* |

Initially, consensus motifs were separated from motif instances using query 1. This statement returned a series of sequence instances in MnM 2 which bind the SH3 domain of a *Target* protein, along with the name of that *Target* protein, e.g.

AKLKPGAPLRPKLN ABL

AKLKPGAPVRSKQL Grb2

AKPKKAPKSPAKA Nck1

By running query 1 again, this time omitting the final 'and' clause, we extract minimotif consensus sequences, where the purpose of the '#' is to format the data on export so that it is directly compatible with the Comparimotif program which was used for comparing instances against consensa (Edwards et al., 2008).

By utilizing the Comparimotif program to compare minimotif instance data against consensa, and integrating this data set to MnM, we could now cross‑query between the results of a global Comparimotif analysis of the motifs using query 2. This revealed the most common SH3 binding motif consensa. This analysis revealed a variety of such relationships between consensus sequences and instances. We ranked relationships by using Comparimotif’s Shannon’s Information Content based score with a cutoff value of 2.0 since low scores did not show meaningful relationships between consensus sequences and instances (Edwards et al., 2008). Considering only scores above this cutoff, we then tabulated a relevance score for important consensus sequences (**Supplementary Table 5**). We define 'Relevance Percent' as the ratio of the number of Comparimotif calculated matches for a consensus by the total amount of distinct instances variants in our database for SH3 binding peptides. For example, a consensus sequence which matched to every SH3 binding instance sequence in MnM 2 would have a score of 100%.

Supplementary Table 4. Consensus sequence relevance ranking.

| **Consensus** | **Relevance Percent** |
| --- | --- |
| Px[IV]PPR | 3.0 |
| PLPxLP | 3.8 |
| [KR]xxxxKx[KR][KR] | 3.8 |
| PxPPxRxSSL | 4.6 |
| RxLPxLP | 4.6 |
| PxPPxRxxSL | 5.2 |
| RxxK | 7.9 |
| KxxK | 8.7 |
| Px[AP]x[PV]R | 22.1 |
| PxLPxK | 12.6 |
| [KR]xLPxxP | 18.8 |
| PxxxPR | 20.7 |
| RxxPxxxP | 24.9 |
| Px[AP]xxR | 33.6 |
| PxxPxK | 35.0 |
| PxxPx[KR] | 74.0 |
| PxxP | 89.1 |

**Supplementary Table 4** indicates importance of all the consensus sequences in the minimotif database in terms of their frequency. The PxxP motif, for example, was an important class since it had the highest frequency. The second most important matches, PxxPx[KR] and PxxPxK are known class II SH3 binding motifs.

Many of the consensus sequences were related as are the two class II motifs above. Therefore we used Cytoscape to visualize all consensus sequences related to instances and grouped motifs that had common sets of instances (Shannon et al., 2003). The visualization of matches using Cytoscape allowed us to identify several important consensus sequences. Although the implementation of Shannon Information Content scoring gives us a valuable initial screen of motif significance, we also used regular expression matching in SQL to identify “exact” matches. Since this was an important query for our analysis, we embedded it in our database as a view (a table with all contents dynamically derived from other tables).

### Supplementary Table 5. Frequencies of exact matching instances / consensus sequences in database.

| **Consensus** | **Number** |
| --- | --- |
| KKPP | 7 |
| PxxxPR | 183 |
| PxxDY | 2 |
| PxxP | 1305 |
| PxxPx[KR] | 972 |
| RxxPxxP | 308 |
| RKxxYxxY | 3 |
| WxxFxLE | 1 |
| [HKR]xxHKR] | 495 |
| KPTVY | 2 |

This analysis resulted in ten different consensus groups (PxxDY, PxxP, [HKR]xx[HKR], PxxxPR, PxxPx[KR], RxxPxxP, WxxxFxxLE, RKxxYxxY, KKPP, and KPTVY). The results from query 3 identified PxxP, RxxPxxP and PxxPx[KR] as the most common motifs (**Supplementary Table 5**). However, PxxxPR, BxxB, and [HKR]xx[HKR] may also be highly significant SH3 binding motifs that bind to distinct sites. Additionally, KKPP, WxxFxxLE, PxxDY, and RKxxYxxY are underrepresented in our database and their broader significance in binding SH3 domains will require further study. One limitation with the frequency‑based analysis is that the SH3 domains and motifs thus far experimentally examined are biased, as may be the content of our database. We have also evaluated the validity of our motif categorization by comparing the binding sites of different SH3 binding motifs in a structural analysis.

## Analysis of residue content in SH3 domain binding peptides

Residue content in all SH3 ligands was determined using queries 4 and 5. Query 5 identifies the frequency of each residue in all SH3 binding minimotifs and these numbers were normalized to the frequency of each residue in the human proteome which was identified using query 5. Query 5 stores this data in a table titled ref_aa_enrichment_human_proteome which has each residue, a percentage value for its enrichment, and its fold enrichment in SH3 binding sequences.
