# Supplementary material for: A proposed syntax for Minimotif Semantics, version 1
Source: BMC Genomics. 2009 Aug 5;10:360. doi: 10.1186/1471-2164-10-360 (PMC2733157; doi:10.1186/1471-2164-10-360)
Supplement: Additional file 2 — Database Documentation files. File of documentation of the MySQL data model. [file 1471-2164-10-360-S2.zip › documentation/Procedures/addKeyPhrase.html]

addKeyPhrase


|  |  |
| --- | --- |
| ``` 155.37.104.15/expertsystem - expertsystem on 155.37.104.15 ``` |  |

addKeyPhrase

Descriptions

There is no description for procedure addKeyPhrase

Parameters

**Name**  **Type**  **Data type** | keyword | In | VARCHAR(255) | | | |

Definition

> ```` ```
> CREATE PROCEDURE `addKeyPhrase`(keyword varchar(255))
>     NOT DETERMINISTIC
>     CONTAINS SQL
>     SQL SECURITY DEFINER
>     COMMENT ''
> insert into ref_pubmedsource_word(word,ref_pubmedsource) select keyword,p.id from ref_pubmedsource p where concat(title,description) like concat('%',keyword,'%');
> ``` ````

---

|  |  |
| --- | --- |
| ``` This file was generated with SQL Manager 2005 for MySQL (www.mysqlmanager.com) at 4/24/2009 1:22 PM ``` |  |
