# Supplementary material for: A proposed syntax for Minimotif Semantics, version 1
Source: BMC Genomics. 2009 Aug 5;10:360. doi: 10.1186/1471-2164-10-360 (PMC2733157; doi:10.1186/1471-2164-10-360)
Supplement: Additional file 2 — Database Documentation files. File of documentation of the MySQL data model. [file 1471-2164-10-360-S2.zip › documentation/Procedures/calculateCorrelation.html]

calculateCorrelation


|  |  |
| --- | --- |
| ``` 155.37.104.15/expertsystem - expertsystem on 155.37.104.15 ``` |  |

calculateCorrelation

Descriptions

There is no description for procedure calculateCorrelation

Parameters

There are no parameters for procedure calculateCorrelation

Definition

> ```` ```
> CREATE PROCEDURE `calculateCorrelation`()
>     NOT DETERMINISTIC
>     CONTAINS SQL
>     SQL SECURITY DEFINER
>     COMMENT ''
> begin
> declare total_p int ;
> declare done int default 0;
> declare total_n int ;
> declare p int default 13;
> declare n int default 15;
> declare w varchar(255) default 'w1';
> declare words cursor for select distinct w.word from ref_pubmedsource_word w where length(w.word) > 1 and not exists (select * from word_score ws where ws.word=w.word);
> declare continue handler for not found set done = 1;
>
> #delete paper scores and word scores... recalculate everything from scratch.
> delete from ref_pubmedsource_score; delete from word_score;
>
> set total_p=(select count(*) from ref_pubmedsource where trainingValue >0);
> set total_n= (select count(*) from ref_pubmedsource where trainingValue <0);
>
>
> open words; 
>
>   REPEAT
>
>     FETCH words INTO w;
>
>     call debugg('starting',concat(w,'  ',p,'  ',n,'  ',total_p,'  ',total_n));
>     commit;
>    #slow
>    # set p = (select count(*) from ref_pubmedsource where description like concat('%',w,'%') and trainingValue>0);
>    # set n = (select count(*) from ref_pubmedsource where description like concat('%',w,'%') and trainingValue<0);
>     
>      set p = (select count(*) from ref_pubmedsource p,ref_pubmedsource_word w1 where word like w and p.id=w1.ref_pubmedsource and trainingValue>0);    
>      set n = (select count(*) from ref_pubmedsource p,ref_pubmedsource_word w1 where word like w and p.id=w1.ref_pubmedsource and trainingValue<0);    
>     
>     replace into word_score values (w,p/total_p-n/total_n);
>
>     call debugg(' done',concat(w,'  p ',p,'  n ',n,'  tp ',total_p,' tn ',total_n));
>     commit; 
>
>     UNTIL done END REPEAT;
>
> #now that scores are calculated, score the papers... 
>   
>   call calculateCorrelation2();
>
> end;
> ``` ````

---

|  |  |
| --- | --- |
| ``` This file was generated with SQL Manager 2005 for MySQL (www.mysqlmanager.com) at 4/24/2009 1:22 PM ``` |  |
