# Supplementary material for: A proposed syntax for Minimotif Semantics, version 1
Source: BMC Genomics. 2009 Aug 5;10:360. doi: 10.1186/1471-2164-10-360 (PMC2733157; doi:10.1186/1471-2164-10-360)
Supplement: Additional file 2 — Database Documentation files. File of documentation of the MySQL data model. [file 1471-2164-10-360-S2.zip › documentation/Procedures/calculateCorrelation2.html]

calculateCorrelation2


|  |  |
| --- | --- |
| ``` 155.37.104.15/expertsystem - expertsystem on 155.37.104.15 ``` |  |

calculateCorrelation2

Descriptions

There is no description for procedure calculateCorrelation2

Parameters

There are no parameters for procedure calculateCorrelation2

Definition

> ```` ```
> CREATE PROCEDURE `calculateCorrelation2`()
>     NOT DETERMINISTIC
>     CONTAINS SQL
>     SQL SECURITY DEFINER
>     COMMENT ''
> BEGIN 
>
> declare done int default 0;
> declare _cid int default 0;
> declare rpms cursor for select distinct id from ref_pubmedsource where score is null and pmid >0 ;
> declare continue handler for not found set done=1;
>
> open rpms;
> #delete from ref_pubmedsource_score;
>
> REPEAT 
>   fetch rpms into _cid;
>
>   delete from debugger;
> commit;
>   call debugg(concat('pubmedsource score',_cid));
>   commit;
>
>
>   update ref_pubmedsource r set score =           
>   ( 
>      select sum(score)
>      from ref_pubmedsource_word w, word_score s      
>      where r.id=_cid
>      and r.id=w.ref_pubmedsource     
>      and  w.word=s.word     
>      group by pmid
>  ) 
>  ;
>
>
>   commit;
>   call debugg(concat('pubmedsource score done...',_cid));
> UNTIL done 
>
> END REPEAT;
>
> END;
> ``` ````

---

|  |  |
| --- | --- |
| ``` This file was generated with SQL Manager 2005 for MySQL (www.mysqlmanager.com) at 4/24/2009 1:22 PM ``` |  |
