# Supplementary material for: A proposed syntax for Minimotif Semantics, version 1
Source: BMC Genomics. 2009 Aug 5;10:360. doi: 10.1186/1471-2164-10-360 (PMC2733157; doi:10.1186/1471-2164-10-360)
Supplement: Additional file 2 — Database Documentation files. File of documentation of the MySQL data model. [file 1471-2164-10-360-S2.zip › documentation/Procedures/debugg.html]

debugg


|  |  |
| --- | --- |
| ``` 155.37.104.15/expertsystem - expertsystem on 155.37.104.15 ``` |  |

debugg

Descriptions

There is no description for procedure debugg

Parameters

**Name**  **Type**  **Data type** | varr | In | LONGTEXT | | | |

Definition

> ```` ```
> CREATE PROCEDURE `debugg`(IN varr LONGTEXT)
>     NOT DETERMINISTIC
>     CONTAINS SQL
>     SQL SECURITY DEFINER
>     COMMENT ''
> BEGIN
>
>      insert into debugger(t) values (varr);
>      commit;
> END;
> ``` ````

---

|  |  |
| --- | --- |
| ``` This file was generated with SQL Manager 2005 for MySQL (www.mysqlmanager.com) at 4/24/2009 1:22 PM ``` |  |
