# Supplementary material for: A proposed syntax for Minimotif Semantics, version 1
Source: BMC Genomics. 2009 Aug 5;10:360. doi: 10.1186/1471-2164-10-360 (PMC2733157; doi:10.1186/1471-2164-10-360)
Supplement: Additional file 2 — Database Documentation files. File of documentation of the MySQL data model. [file 1471-2164-10-360-S2.zip › documentation/Procedures/domainsGroupedBy.html]

domainsGroupedBy


|  |  |
| --- | --- |
| ``` 155.37.104.15/expertsystem - expertsystem on 155.37.104.15 ``` |  |

domainsGroupedBy

Descriptions

There is no description for procedure domainsGroupedBy

Parameters

There are no parameters for procedure domainsGroupedBy

Definition

> ```` ```
> CREATE PROCEDURE `domainsGroupedBy`()
>     NOT DETERMINISTIC
>     CONTAINS SQL
>     SQL SECURITY DEFINER
>     COMMENT ''
> BEGIN
>
> select
> m.sequence regexp('B..B') as 'bxxb',
> m.sequence regexp('P..P') as 'pxxp',
> activityClass,
> d.domain,
> count(*),
> avg(getPeptideCharge(m.sequence)),
> count(*),
> stddev(getPeptideCharge(m.sequence)),
> t.name
>
> from
>
> motif m,
> motif_source s,
> ref_knownactivity a,
> ref_molecule t,
> ref_domain d
>
> where
>
> a.id=s.knownActivity
> and
> m.id=s.motif
> and
> s.target=t.id
> and
> t.ref_domain=d.id
>
> group by s.knownactivity, d.domain, m.sequence regexp('B..B')
>
> order by count(*);
>
> END;
> ``` ````

---

|  |  |
| --- | --- |
| ``` This file was generated with SQL Manager 2005 for MySQL (www.mysqlmanager.com) at 4/24/2009 1:22 PM ``` |  |
