# Supplementary material for: A proposed syntax for Minimotif Semantics, version 1
Source: BMC Genomics. 2009 Aug 5;10:360. doi: 10.1186/1471-2164-10-360 (PMC2733157; doi:10.1186/1471-2164-10-360)
Supplement: Additional file 2 — Database Documentation files. File of documentation of the MySQL data model. [file 1471-2164-10-360-S2.zip › documentation/Procedures/generateSpreadsheet.html]

generateSpreadsheet


|  |  |
| --- | --- |
| ``` 155.37.104.15/expertsystem - expertsystem on 155.37.104.15 ``` |  |

generateSpreadsheet

Descriptions

There is no description for procedure generateSpreadsheet

Parameters

There are no parameters for procedure generateSpreadsheet

Definition

> ```` ```
> CREATE PROCEDURE `generateSpreadsheet`()
>     DETERMINISTIC
>     CONTAINS SQL
>     SQL SECURITY DEFINER
>     COMMENT ''
> BEGIN
>
>  SELECT
>    *
>   FROM
>        motif_source ms
>   	  LEFT OUTER JOIN ref_molecule src on src.id=ms.`motifProtein`    
>   	  LEFT OUTER JOIN ref_molecule tgt on tgt.id=ms.`target`
> 	  LEFT OUTER JOIN ref_knownActivity a on a.id=ms.`knownActivity`
>        	  LEFT OUTER JOIN ref_domain d on d.id=tgt.ref_domain
>       	  LEFT OUTER JOIN motif m on m.id=ms.motif
>   
>
> ;
> END;
> ``` ````

---

|  |  |
| --- | --- |
| ``` This file was generated with SQL Manager 2005 for MySQL (www.mysqlmanager.com) at 4/24/2009 1:22 PM ``` |  |
