# Supplementary material for: A proposed syntax for Minimotif Semantics, version 1
Source: BMC Genomics. 2009 Aug 5;10:360. doi: 10.1186/1471-2164-10-360 (PMC2733157; doi:10.1186/1471-2164-10-360)
Supplement: Additional file 2 — Database Documentation files. File of documentation of the MySQL data model. [file 1471-2164-10-360-S2.zip › documentation/Procedures/getMotifsByProperty.html]

getMotifsByProperty


|  |  |
| --- | --- |
| ``` 155.37.104.15/expertsystem - expertsystem on 155.37.104.15 ``` |  |

getMotifsByProperty

Descriptions

There is no description for procedure getMotifsByProperty

Parameters

**Name**  **Type**  **Data type** | imotif | In | VARCHAR(255) | | iactivityClass | In | VARCHAR(255) | | iactivitySubclass | In | VARCHAR(255) | | itargetDomain | In | VARCHAR(255) | | itargetName | In | VARCHAR(255) | | | |

Definition

> ```` ```
> CREATE PROCEDURE `getMotifsByProperty`(imotif varchar(255), iactivityClass varchar(255), iactivitySubclass varchar(255), itargetDomain varchar(255), itargetName varchar(255))
>     NOT DETERMINISTIC
>     CONTAINS SQL
>     SQL SECURITY DEFINER
>     COMMENT ''
> BEGIN
> 	SELECT
>   	  ms.id as id,
>   	  m.sequence as sequence, 
>   	  tgt.name as targetName, 
>   	  src.name as proteinName, 
>   	  activityClass as activityClass, 
>   	  activitySubclass as activitySubclass, 
>   	  d.domain as targetDomain
>   	FROM
>       motif_source ms
>   	  LEFT OUTER JOIN ref_molecule src on src.id=ms.`motifProtein`    
>   	  LEFT OUTER JOIN ref_molecule tgt on tgt.id=ms.`target`
> 	  LEFT OUTER JOIN ref_knownActivity a on a.id=ms.`knownActivity`
>       LEFT OUTER JOIN ref_domain d on d.id=tgt.ref_domain
>       LEFT OUTER JOIN motif m on m.id=ms.motif
>   	WHERE sequence regexp(imotif)
>   	AND (activityClass like (iactivityClass) or length(iactivityClass)=0)
>   	AND (activitySubclass like (iactivitySubclass) OR length(activitySubclass)=0)
>   	AND (d.domain like (itargetDomain) OR length(itargetDomain)=0)
>   	AND (tgt.name like (itargetName) OR length(itargetName)=0)
> 	limit 100
> ;
> END;
> ``` ````

---

|  |  |
| --- | --- |
| ``` This file was generated with SQL Manager 2005 for MySQL (www.mysqlmanager.com) at 4/24/2009 1:22 PM ``` |  |
