# Supplementary material for: A proposed syntax for Minimotif Semantics, version 1
Source: BMC Genomics. 2009 Aug 5;10:360. doi: 10.1186/1471-2164-10-360 (PMC2733157; doi:10.1186/1471-2164-10-360)
Supplement: Additional file 2 — Database Documentation files. File of documentation of the MySQL data model. [file 1471-2164-10-360-S2.zip › documentation/Procedures/Index.html]

Procedures


|  |  |
| --- | --- |
| ``` 155.37.104.15/expertsystem - expertsystem on 155.37.104.15 ``` |  |

Procedures

**Procedure**  **Type**  **SQL Security**  **Description** | addKeyPhrase | Not deterministic | Definer |  | | calculateCorrelation | Not deterministic | Definer |  | | calculateCorrelation2 | Not deterministic | Definer |  | | debugg | Not deterministic | Definer |  | | domainsGroupedBy | Not deterministic | Definer |  | | generateSpreadsheet | Deterministic | Definer |  | | getMotifsByProperty | Not deterministic | Definer |  | | quickies | Deterministic | Definer |  | | update\_motif\_modifications | Not deterministic | Definer |  | | update\_ref\_molecule\_species\_names | Not deterministic | Definer |  | | | | |

---

|  |  |
| --- | --- |
| ``` This file was generated with SQL Manager 2005 for MySQL (www.mysqlmanager.com) at 4/24/2009 1:22 PM ``` |  |
