# Supplementary material for: A proposed syntax for Minimotif Semantics, version 1
Source: BMC Genomics. 2009 Aug 5;10:360. doi: 10.1186/1471-2164-10-360 (PMC2733157; doi:10.1186/1471-2164-10-360)
Supplement: Additional file 2 — Database Documentation files. File of documentation of the MySQL data model. [file 1471-2164-10-360-S2.zip › documentation/Procedures/quickies.html]

quickies


|  |  |
| --- | --- |
| ``` 155.37.104.15/expertsystem - expertsystem on 155.37.104.15 ``` |  |

quickies

Descriptions

There is no description for procedure quickies

Parameters

There are no parameters for procedure quickies

Definition

> ```` ```
> CREATE PROCEDURE `quickies`()
>     DETERMINISTIC
>     CONTAINS SQL
>     SQL SECURITY DEFINER
>     COMMENT ''
> begin
>
>
> update motif set mtype='Consensus' where mtype like '%nsus%';
>
> end;
> ``` ````

---

|  |  |
| --- | --- |
| ``` This file was generated with SQL Manager 2005 for MySQL (www.mysqlmanager.com) at 4/24/2009 1:22 PM ``` |  |
