# Supplementary material for: A proposed syntax for Minimotif Semantics, version 1
Source: BMC Genomics. 2009 Aug 5;10:360. doi: 10.1186/1471-2164-10-360 (PMC2733157; doi:10.1186/1471-2164-10-360)
Supplement: Additional file 2 — Database Documentation files. File of documentation of the MySQL data model. [file 1471-2164-10-360-S2.zip › documentation/Procedures/update_ref_molecule_species_names.html]

update\_ref\_molecule\_species\_names


|  |  |
| --- | --- |
| ``` 155.37.104.15/expertsystem - expertsystem on 155.37.104.15 ``` |  |

update\_ref\_molecule\_species\_names

Descriptions

There is no description for procedure update\_ref\_molecule\_species\_names

Parameters

There are no parameters for procedure update\_ref\_molecule\_species\_names

Definition

> ```` ```
> CREATE PROCEDURE `update_ref_molecule_species_names`()
>     NOT DETERMINISTIC
>     CONTAINS SQL
>     SQL SECURITY DEFINER
>     COMMENT ''
> BEGIN
>
> 	update ref_molecule m set species = 
> 	( 
> 		select taxid from ncbi_federated.RefseqEntry  where trim(m.dbIdentifier)=rsid limit 1
> 	);
>  	update ref_molecule m set speciesName = 
> 	( 
> 		select taxonomy from ncbi_federated.RefseqEntry  where trim(m.dbIdentifier)=rsid limit 1
> 	);
> 	commit;
>
> update ref_molecule set speciesName = null where species = 0;
>
> select count(*) from ref_molecule where not species=0 
> union select count(*) from ref_molecule where not  (speciesName is null) ;
>
> END;
> ``` ````

---

|  |  |
| --- | --- |
| ``` This file was generated with SQL Manager 2005 for MySQL (www.mysqlmanager.com) at 4/24/2009 1:22 PM ``` |  |
