# Supplementary material for: A proposed syntax for Minimotif Semantics, version 1
Source: BMC Genomics. 2009 Aug 5;10:360. doi: 10.1186/1471-2164-10-360 (PMC2733157; doi:10.1186/1471-2164-10-360)
Supplement: Additional file 2 — Database Documentation files. File of documentation of the MySQL data model. [file 1471-2164-10-360-S2.zip › documentation/Tables/domainproteinpaper.html]

domainproteinpaper


|  |  |
| --- | --- |
| ``` 155.37.104.15/expertsystem - expertsystem on 155.37.104.15 ``` |  |

domainproteinpaper

Descriptions

There is no description for table domainproteinpaper

Fields

**PK**  **Name**  **Data type**  **Size**  **Precision**  **Values**  **Default**  **Auto Increment**  **Binary**  **Not null**  **Unsigned**  **Zero Fill**  **Unique** |  | rd\_id | INTEGER | 11 | 0 |  |  |  |  |  |  |  |  | |  | rpms\_id | INTEGER | 11 | 0 |  |  |  |  |  |  |  |  | |  | rpp\_id | INTEGER | 11 | 0 |  |  |  |  |  |  |  |  | |  | rpd\_id | INTEGER | 11 | 0 |  |  |  |  |  |  |  |  | |  | protein | LONGTEXT | 0 | 0 |  |  |  |  |  |  |  |  | |  | domain | VARCHAR | 255 | 0 |  |  |  |  |  |  |  |  | | | | | | | | | | | | | |

Indices

There are no indices for table domainproteinpaper

Triggers

There are no triggers for table domainproteinpaper

Options

**TransactSafe**  **TableType**  **Row Format**  **Check Sum**  **Delay Key Write**  **Pack Keys**  **Temporary**  **Min Rows**  **Max Rows**  **Union** |  | MyISAM | DYNAMIC |  |  |  |  | 0 | 0 |  | | | | | | | | | | |

Definition

> ```` ```
> CREATE TABLE `domainproteinpaper` (
>   `rd_id` int(11) default NULL,
>   `rpms_id` int(11) default NULL,
>   `rpp_id` int(11) default NULL,
>   `rpd_id` int(11) default NULL,
>   `protein` longtext,
>   `domain` varchar(255) default NULL
> ) ENGINE=MyISAM DEFAULT CHARSET=latin1;
> ``` ````

---

|  |  |
| --- | --- |
| ``` This file was generated with SQL Manager 2005 for MySQL (www.mysqlmanager.com) at 4/24/2009 1:22 PM ``` |  |
