# Supplementary material for: A proposed syntax for Minimotif Semantics, version 1
Source: BMC Genomics. 2009 Aug 5;10:360. doi: 10.1186/1471-2164-10-360 (PMC2733157; doi:10.1186/1471-2164-10-360)
Supplement: Additional file 2 — Database Documentation files. File of documentation of the MySQL data model. [file 1471-2164-10-360-S2.zip › documentation/Tables/Index.html]

Tables


|  |  |
| --- | --- |
| ``` 155.37.104.15/expertsystem - expertsystem on 155.37.104.15 ``` |  |

Tables

**Table**  **Type**  **Cols**  **Rows**  **Create Time**  **Update Time**  **Check Time**  **Description** | annotation\_rule | InnoDB | 2 | 20 | 4/23/2009 1:53 PM |  |  | InnoDB free: 31744 kB | | comparimotif\_sh3 | InnoDB | 3 | 2119 | 4/21/2009 9:35 AM |  |  | InnoDB free: 31744 kB | | debugger | MyISAM | 2 | 1 | 3/13/2009 3:29 PM | 3/13/2009 3:29 PM |  |  | | distinct\_sh3\_binding\_lexica\_types | MyISAM | 6 | 0 | 3/13/2009 3:29 PM | 3/13/2009 3:29 PM |  |  | | domainproteinpaper | MyISAM | 6 | 0 | 3/13/2009 3:29 PM | 3/13/2009 3:29 PM |  |  | | highlight | InnoDB | 3 | 4 | 3/13/2009 3:29 PM |  |  | InnoDB free: 31744 kB | | highlight\_exclude | InnoDB | 2 | 4 | 3/13/2009 3:29 PM |  |  | InnoDB free: 31744 kB | | human\_proteome | MyISAM | 3 | 0 | 3/13/2009 3:29 PM | 3/13/2009 3:29 PM |  |  | | join\_motif\_src\_target | MyISAM | 5 | 0 | 3/13/2009 3:29 PM | 3/13/2009 3:29 PM |  |  | | motif | InnoDB | 6 | 2461 | 4/16/2009 3:05 PM |  |  | InnoDB free: 31744 kB | | motif\_group | InnoDB | 2 | 11 | 3/13/2009 3:29 PM |  |  | InnoDB free: 31744 kB | | motif\_modification | InnoDB | 6 | 730 | 4/23/2009 5:07 PM |  |  | InnoDB free: 31744 kB; (`motif\_source`) REFER `expertsystem/motif\_source`(`id`) | | motif\_source | InnoDB | 19 | 5697 | 4/14/2009 2:34 PM |  |  | InnoDB free: 31744 kB; (`knownActivity`) REFER `expertsystem/ref\_knownactivity`( | | motif\_source\_motif\_group | InnoDB | 3 | 1987 | 3/13/2009 3:29 PM |  |  | InnoDB free: 31744 kB | | motif\_source\_pubmedsource | InnoDB | 3 | 4864 | 4/3/2009 12:24 PM |  |  | InnoDB free: 31744 kB; (`ref\_pubmedsource`) REFER `expertsystem/ref\_pubmedsource | | motif\_source\_xref2 | InnoDB | 4 | 1131 | 4/6/2009 9:14 PM |  |  | InnoDB free: 31744 kB | | mymfavorites | InnoDB | 4 | 1 | 3/13/2009 3:29 PM |  |  | Please do not modify this table!; InnoDB free: 31744 kB | | new\_view | MyISAM | 2 | 0 | 3/13/2009 3:29 PM | 3/13/2009 3:29 PM |  |  | | nonmotifs | InnoDB | 1 | 0 | 3/13/2009 3:29 PM |  |  | InnoDB free: 31744 kB | | pdz\_comparison | MyISAM | 5 | 0 | 3/13/2009 3:29 PM | 3/13/2009 3:29 PM |  |  | | proteinbin | InnoDB | 5 | 3754 | 3/13/2009 3:29 PM |  |  | InnoDB free: 31744 kB; (`refDomain`) REFER `expertsystem/ref\_domain`(`id`); (`pr | | pubmedsource\_reviewevent | InnoDB | 5 | 146018 | 4/3/2009 12:24 PM |  |  | InnoDB free: 31744 kB; (`pubmedsource\_id`) REFER `expertsystem/ref\_pubmedsource` | | ref\_aa\_enrichment\_human\_proteome | InnoDB | 2 | 21 | 3/13/2009 3:29 PM |  |  | InnoDB free: 31744 kB | | ref\_amino\_acid | InnoDB | 1 | 21 | 3/13/2009 3:29 PM |  |  | InnoDB free: 31744 kB | | ref\_cellcompartment | InnoDB | 2 | 275 | 3/13/2009 3:29 PM |  |  | InnoDB free: 31744 kB | | ref\_completed\_species | InnoDB | 3 | 77 | 3/13/2009 3:29 PM |  |  | InnoDB free: 31744 kB | | ref\_domain | InnoDB | 2 | 10987 | 3/13/2009 3:29 PM |  |  | InnoDB free: 31744 kB | | ref\_homologene | InnoDB | 7 | 179624 | 3/13/2009 3:29 PM |  |  | InnoDB free: 31744 kB | | ref\_homologene\_2 | InnoDB | 4 | 48543 | 3/13/2009 3:29 PM |  |  | InnoDB free: 2550784 kB; InnoDB free: 31744 kB | | ref\_homologene\_2\_alias | InnoDB | 3 | 243882 | 3/13/2009 3:30 PM |  |  | InnoDB free: 31744 kB; (`ref\_homologene\_2\_gene`) REFER `expertsystem/ref\_homolog | | ref\_homologene\_2\_gene | InnoDB | 11 | 208647 | 3/13/2009 3:30 PM |  |  | InnoDB free: 31744 kB; (`ref\_homologene\_2`) REFER `expertsystem/ref\_homologene\_2 | | ref\_homologene\_2\_gene\_domain | InnoDB | 5 | 276863 | 3/13/2009 3:31 PM |  |  | InnoDB free: 31744 kB; (`ref\_homologene\_gene`) REFER `expertsystem/ref\_homologen | | ref\_homologene\_2\_gene\_protein | InnoDB | 8 | 4418412 | 3/13/2009 3:32 PM |  |  | InnoDB free: 31744 kB; (`ref\_homologene\_2\_gene`) REFER `expertsystem/ref\_homolog | | ref\_homologene\_2\_taxonomy | InnoDB | 3 | 535170 | 3/13/2009 3:49 PM |  |  | InnoDB free: 31744 kB | | ref\_knownactivity | InnoDB | 7 | 97 | 4/15/2009 7:15 PM |  |  | InnoDB free: 159744 kB; InnoDB free: 31744 kB | | ref\_knowntechnique | InnoDB | 6 | 23 | 4/14/2009 2:34 PM |  |  | InnoDB free: 31744 kB | | ref\_knowntechnique\_xref | InnoDB | 4 | 22 | 4/14/2009 2:34 PM |  |  | InnoDB free: 31744 kB | | ref\_levenstein\_map | InnoDB | 3 | 1750494 | 3/13/2009 3:49 PM |  |  | InnoDB free: 31744 kB | | ref\_molecule | InnoDB | 11 | 8727 | 4/16/2009 3:44 PM |  |  | InnoDB free: 31744 kB; (`ref\_domain`) REFER `expertsystem/ref\_domain`(`id`); (`r | | ref\_motif\_distance | InnoDB | 3 | 0 | 3/13/2009 3:50 PM |  |  | InnoDB free: 31744 kB | | ref\_motifcomparison | InnoDB | 11 | 23147 | 3/13/2009 3:50 PM |  |  | InnoDB free: 31744 kB | | ref\_pms\_domain | InnoDB | 3 | 81467 | 3/13/2009 3:50 PM |  |  | InnoDB free: 31744 kB; (`refdomain`) REFER `expertsystem/ref\_domain`(`id`); (`pm | | ref\_pms\_protein | InnoDB | 4 | 1104732 | 3/13/2009 3:50 PM |  |  | InnoDB free: 31744 kB; (`ref\_homologene\_2\_gene\_protein`) REFER `expertsystem/ref | | ref\_pms\_protein\_tag | InnoDB | 4 | 1204691 | 3/13/2009 4:00 PM |  |  | InnoDB free: 31744 kB; (`ref\_pms\_protein`) REFER `expertsystem/ref\_pms\_protein`( | | ref\_pmsusertag | InnoDB | 4 | 24 | 3/13/2009 4:04 PM |  |  | InnoDB free: 31744 kB; (`ref\_pubmedsource`) REFER `expertsystem/ref\_pubmedsource | | ref\_pubmed\_query | InnoDB | 2 | 62 | 3/13/2009 4:04 PM |  |  | InnoDB free: 31744 kB | | ref\_pubmed\_query\_result | InnoDB | 3 | 456594 | 3/13/2009 4:04 PM |  |  | InnoDB free: 31744 kB; (`pms`) REFER `expertsystem/ref\_pubmedsource`(`id`); (`qu | | ref\_pubmedsource | InnoDB | 16 | 125356 | 4/3/2009 12:25 PM |  |  | InnoDB free: 31744 kB | | ref\_pubmedsource\_common\_words | InnoDB | 2 | 503 | 3/13/2009 4:09 PM |  |  | InnoDB free: 31744 kB | | ref\_pubmedsource\_score | MyISAM | 2 | 108153 | 3/13/2009 4:09 PM | 3/13/2009 4:09 PM |  |  | | ref\_pubmedsource\_word | InnoDB | 3 | 1133343 | 3/13/2009 4:09 PM |  |  | InnoDB free: 31744 kB; (`ref\_pubmedsource`) REFER `expertsystem/ref\_pubmedsource | | ref\_pubmedsource\_word\_abridged | MyISAM | 3 | 0 | 3/13/2009 3:29 PM | 3/13/2009 3:29 PM |  |  | | reviewevent | InnoDB | 7 | 5318 | 4/14/2009 2:34 PM |  |  | InnoDB free: 31744 kB | | sh3\_binding\_motif\_classes | MyISAM | 2 | 0 | 3/13/2009 3:29 PM | 3/13/2009 3:29 PM |  |  | | sh3\_binding\_motif\_groups\_delete\_me | MyISAM | 5 | 0 | 3/13/2009 3:29 PM | 3/13/2009 3:29 PM |  |  | | sh3\_binding\_motifs\_sandbox | MyISAM | 6 | 0 | 3/13/2009 3:29 PM | 3/13/2009 3:29 PM |  |  | | spreadsheet | MyISAM | 6 | 0 | 3/13/2009 3:29 PM | 3/13/2009 3:29 PM |  |  | | technique | InnoDB | 4 | 3594 | 4/3/2009 12:24 PM |  |  | InnoDB free: 31744 kB; (`motifsource`) REFER `expertsystem/motif\_source`(`id`); | | test\_reviewevents | MyISAM | 5 | 0 | 3/13/2009 3:29 PM | 3/13/2009 3:29 PM |  |  | | test\_reviewevents\_2 | MyISAM | 3 | 0 | 3/13/2009 3:29 PM | 3/13/2009 3:29 PM |  |  | | user | InnoDB | 6 | 8 | 4/3/2009 12:24 PM |  |  | InnoDB free: 31744 kB | | word\_score | MyISAM | 2 | 0 | 4/6/2009 9:52 PM | 4/6/2009 9:52 PM |  |  | | | | | | | | |

---

|  |  |
| --- | --- |
| ``` This file was generated with SQL Manager 2005 for MySQL (www.mysqlmanager.com) at 4/24/2009 1:22 PM ``` |  |
