# Supplementary material for: A proposed syntax for Minimotif Semantics, version 1
Source: BMC Genomics. 2009 Aug 5;10:360. doi: 10.1186/1471-2164-10-360 (PMC2733157; doi:10.1186/1471-2164-10-360)
Supplement: Additional file 2 — Database Documentation files. File of documentation of the MySQL data model. [file 1471-2164-10-360-S2.zip › documentation/Tables/motif_source.html]

motif\_source


|  |  |
| --- | --- |
| ``` 155.37.104.15/expertsystem - expertsystem on 155.37.104.15 ``` |  |

motif\_source

Descriptions

InnoDB free: 31744 kB; (`knownActivity`) REFER `expertsystem/ref\_knownactivity`(

Fields

**PK**  **Name**  **Data type**  **Size**  **Precision**  **Values**  **Default**  **Auto Increment**  **Binary**  **Not null**  **Unsigned**  **Zero Fill**  **Unique** |  | id | INTEGER | 11 | 0 |  |  |  |  |  |  |  |  | |  | subcellularLocalization | VARCHAR | 255 | 0 |  |  |  |  |  |  |  |  | |  | knownActivity | INTEGER | 11 | 0 |  |  |  |  |  |  |  |  | |  | motifProtein | INTEGER | 11 | 0 |  |  |  |  |  |  |  |  | |  | target | INTEGER | 11 | 0 |  |  |  |  |  |  |  |  | |  | microMolarAffinity | FLOAT | 0 | 0 |  |  |  |  |  |  |  |  | |  | relatedStructures | TEXT | 0 | 0 |  |  |  |  |  |  |  |  | |  | pdbid | TEXT | 0 | 0 |  |  |  |  |  |  |  |  | |  | motif | INTEGER | 11 | 0 |  |  |  |  |  |  |  |  | |  | reviewevent | INTEGER | 11 | 0 |  | -1 |  |  |  |  |  |  | |  | targetdomain | INTEGER | 11 | 0 |  |  |  |  |  |  |  |  | |  | traffickingFunction | VARCHAR | 255 | 0 |  |  |  |  |  |  |  |  | |  | multidomain | INTEGER | 5 | 0 |  |  |  |  |  |  |  |  | |  | cellDomain | VARCHAR | 255 | 0 |  |  |  |  |  |  |  |  | |  | targetSite | INTEGER | 11 | 0 |  |  |  |  |  |  |  |  | |  | complex | TINYINT | 1 | 0 |  |  |  |  |  |  |  |  | |  | affinityrange | FLOAT | 0 | 0 |  |  |  |  |  |  |  |  | |  | comment | LONGTEXT | 0 | 0 |  |  |  |  |  |  |  |  | |  | knownActivityNormalized | INTEGER | 11 | 0 |  |  |  |  |  |  |  |  | | | | | | | | | | | | | |

Indices

**Name**  **Fields**  **Unique**  **Collation**  **Full Text** | PRIMARY | id |  | Ascending |  | | subcellularLocalization | subcellularLocalization |  | Ascending |  | | knownActivity | knownActivity |  | Ascending |  | | motifProtein | motifProtein |  | Ascending |  | | motifsource2\_fk3 | target |  | Ascending |  | | motif | motif |  | Ascending |  | | reviewevent | reviewevent |  | Ascending |  | | targetdomain | targetdomain |  | Ascending |  | | | | | |

Foreign Keys

**Fields**  **Foreign Database**  **Foreign Table**  **Foreign Fields**  **Update Action**  **Delete Action** | `knownActivity` | expertsystem | ref\_knownactivity | `id` | Restrict | Restrict | | `motifProtein` | expertsystem | ref\_molecule | `id` | Restrict | Restrict | | `motif` | expertsystem | motif | `id` | Restrict | Restrict | | `targetdomain` | expertsystem | ref\_domain | `id` | Restrict | Restrict | | `subcellularLocalization` | expertsystem | ref\_cellcompartment | `cname` | Restrict | Restrict | | | | | | |

Triggers

There are no triggers for table motif\_source

Options

**TransactSafe**  **TableType**  **Row Format**  **Check Sum**  **Delay Key Write**  **Pack Keys**  **Temporary**  **Min Rows**  **Max Rows**  **Union** |  | InnoDB | Ascending |  |  |  |  | 0 | 0 |  | | | | | | | | | | |

Definition

> ```` ```
> CREATE TABLE `motif_source` (
>   `id` int(11) NOT NULL auto_increment,
>   `subcellularLocalization` varchar(255) default NULL,
>   `knownActivity` int(11) default NULL,
>   `motifProtein` int(11) default NULL,
>   `target` int(11) default NULL,
>   `microMolarAffinity` float default NULL,
>   `relatedStructures` text,
>   `pdbid` text,
>   `motif` int(11) NOT NULL,
>   `reviewevent` int(11) NOT NULL default '-1',
>   `targetdomain` int(11) default NULL,
>   `traffickingFunction` varchar(255) default NULL,
>   `multidomain` int(5) default NULL,
>   `cellDomain` varchar(255) default NULL,
>   `targetSite` int(11) default NULL,
>   `complex` tinyint(1) default NULL,
>   `affinityrange` float default NULL,
>   `comment` longtext,
>   `knownActivityNormalized` int(11) NOT NULL,
>   PRIMARY KEY  (`id`),
>   KEY `subcellularLocalization` (`subcellularLocalization`),
>   KEY `knownActivity` (`knownActivity`),
>   KEY `motifProtein` (`motifProtein`),
>   KEY `motifsource2_fk3` (`target`),
>   KEY `motif` (`motif`),
>   KEY `reviewevent` (`reviewevent`),
>   KEY `targetdomain` (`targetdomain`),
>   CONSTRAINT `motifsource2_fk1` FOREIGN KEY (`knownActivity`) REFERENCES `ref_knownactivity` (`id`),
>   CONSTRAINT `motifsource2_fk2` FOREIGN KEY (`motifProtein`) REFERENCES `ref_molecule` (`id`),
>   CONSTRAINT `motifsource2_fk4` FOREIGN KEY (`motif`) REFERENCES `motif` (`id`),
>   CONSTRAINT `motif_source_fk` FOREIGN KEY (`targetdomain`) REFERENCES `ref_domain` (`id`),
>   CONSTRAINT `motif_source_fk1` FOREIGN KEY (`subcellularLocalization`) REFERENCES `ref_cellcompartment` (`cname`)
> ) ENGINE=InnoDB AUTO_INCREMENT=70632 DEFAULT CHARSET=latin1;
> ``` ````

---

|  |  |
| --- | --- |
| ``` This file was generated with SQL Manager 2005 for MySQL (www.mysqlmanager.com) at 4/24/2009 1:22 PM ``` |  |
