# Supplementary material for: A proposed syntax for Minimotif Semantics, version 1
Source: BMC Genomics. 2009 Aug 5;10:360. doi: 10.1186/1471-2164-10-360 (PMC2733157; doi:10.1186/1471-2164-10-360)
Supplement: Additional file 2 — Database Documentation files. File of documentation of the MySQL data model. [file 1471-2164-10-360-S2.zip › documentation/Tables/ref_pubmed_query_result.html]

ref\_pubmed\_query\_result


|  |  |
| --- | --- |
| ``` 155.37.104.15/expertsystem - expertsystem on 155.37.104.15 ``` |  |

ref\_pubmed\_query\_result

Descriptions

InnoDB free: 31744 kB; (`pms`) REFER `expertsystem/ref\_pubmedsource`(`id`); (`qu

Fields

**PK**  **Name**  **Data type**  **Size**  **Precision**  **Values**  **Default**  **Auto Increment**  **Binary**  **Not null**  **Unsigned**  **Zero Fill**  **Unique** |  | id | INTEGER | 11 | 0 |  |  |  |  |  |  |  |  | |  | query | INTEGER | 11 | 0 |  |  |  |  |  |  |  |  | |  | pms | INTEGER | 11 | 0 |  |  |  |  |  |  |  |  | | | | | | | | | | | | | |

Indices

**Name**  **Fields**  **Unique**  **Collation**  **Full Text** | PRIMARY | id |  | Ascending |  | | pms | pms |  | Ascending |  | | query | query |  | Ascending |  | | | | | |

Foreign Keys

**Fields**  **Foreign Database**  **Foreign Table**  **Foreign Fields**  **Update Action**  **Delete Action** | `pms` | expertsystem | ref\_pubmedsource | `id` | Restrict | Restrict | | `query` | expertsystem | ref\_pubmed\_query | `id` | Restrict | Restrict | | | | | | |

Triggers

There are no triggers for table ref\_pubmed\_query\_result

Options

**TransactSafe**  **TableType**  **Row Format**  **Check Sum**  **Delay Key Write**  **Pack Keys**  **Temporary**  **Min Rows**  **Max Rows**  **Union** |  | InnoDB | Ascending |  |  |  |  | 0 | 0 |  | | | | | | | | | | |

Definition

> ```` ```
> CREATE TABLE `ref_pubmed_query_result` (
>   `id` int(11) NOT NULL auto_increment,
>   `query` int(11) NOT NULL,
>   `pms` int(11) NOT NULL,
>   PRIMARY KEY  (`id`),
>   KEY `pms` (`pms`),
>   KEY `query` (`query`),
>   CONSTRAINT `ref_pubmed_query_result_fk` FOREIGN KEY (`pms`) REFERENCES `ref_pubmedsource` (`id`),
>   CONSTRAINT `ref_pubmed_query_result_fk1` FOREIGN KEY (`query`) REFERENCES `ref_pubmed_query` (`id`)
> ) ENGINE=InnoDB AUTO_INCREMENT=477149 DEFAULT CHARSET=latin1;
> ``` ````

---

|  |  |
| --- | --- |
| ``` This file was generated with SQL Manager 2005 for MySQL (www.mysqlmanager.com) at 4/24/2009 1:22 PM ``` |  |
