# Supplementary material for: A proposed syntax for Minimotif Semantics, version 1
Source: BMC Genomics. 2009 Aug 5;10:360. doi: 10.1186/1471-2164-10-360 (PMC2733157; doi:10.1186/1471-2164-10-360)
Supplement: Additional file 2 — Database Documentation files. File of documentation of the MySQL data model. [file 1471-2164-10-360-S2.zip › documentation/Tables/ref_pubmedsource.html]

ref\_pubmedsource


|  |  |
| --- | --- |
| ``` 155.37.104.15/expertsystem - expertsystem on 155.37.104.15 ``` |  |

ref\_pubmedsource

Descriptions

InnoDB free: 31744 kB

Fields

**PK**  **Name**  **Data type**  **Size**  **Precision**  **Values**  **Default**  **Auto Increment**  **Binary**  **Not null**  **Unsigned**  **Zero Fill**  **Unique** |  | description | LONGTEXT | 0 | 0 |  |  |  |  |  |  |  |  | |  | type | VARCHAR | 40 | 0 |  |  |  |  |  |  |  |  | |  | id | INTEGER | 11 | 0 |  |  |  |  |  |  |  |  | |  | pmid | INTEGER | 11 | 0 |  |  |  |  |  |  |  |  | |  | url | LONGTEXT | 0 | 0 |  |  |  |  |  |  |  |  | |  | isComplete | TINYINT | 1 | 0 |  | 1 |  |  |  |  |  |  | |  | title | LONGTEXT | 0 | 0 |  |  |  |  |  |  |  |  | |  | searchdescription | LONGTEXT | 0 | 0 |  |  |  |  |  |  |  |  | |  | affiliation | VARCHAR | 255 | 0 |  |  |  |  |  |  |  |  | |  | journal | VARCHAR | 255 | 0 |  |  |  |  |  |  |  |  | |  | author | VARCHAR | 255 | 0 |  |  |  |  |  |  |  |  | |  | date | VARCHAR | 255 | 0 |  |  |  |  |  |  |  |  | |  | expectedMotifCount | INTEGER | 11 | 0 |  | 1 |  |  |  |  |  |  | |  | tracking\_status | VARCHAR | 255 | 0 |  |  |  |  |  |  |  |  | |  | comment | LONGTEXT | 0 | 0 |  |  |  |  |  |  |  |  | |  | score | FLOAT | 0 | 0 |  |  |  |  |  |  |  |  | | | | | | | | | | | | | |

Indices

**Name**  **Fields**  **Unique**  **Collation**  **Full Text** | PRIMARY | id |  | Ascending |  | | pmid | pmid |  | Ascending |  | | pmid\_2 | pmid |  | Ascending |  | | journal\_a | journal |  | Ascending |  | | journal\_d | journal |  | Ascending |  | | date\_a | date |  | Ascending |  | | date\_d | date |  | Ascending |  | | trackingStatus\_a | tracking\_status |  | Ascending |  | | trackingStatus\_d | tracking\_status |  | Ascending |  | | pmid\_a | pmid |  | Ascending |  | | pmid\_d | pmid |  | Ascending |  | | id\_a | id |  | Ascending |  | | | | | |

Foreign Keys

There are no foreign keys for table ref\_pubmedsource

Triggers

There are no triggers for table ref\_pubmedsource

Options

**TransactSafe**  **TableType**  **Row Format**  **Check Sum**  **Delay Key Write**  **Pack Keys**  **Temporary**  **Min Rows**  **Max Rows**  **Union** |  | InnoDB | Ascending |  |  |  |  | 0 | 0 |  | | | | | | | | | | |

Definition

> ```` ```
> CREATE TABLE `ref_pubmedsource` (
>   `description` longtext,
>   `type` varchar(40) default NULL,
>   `id` int(11) NOT NULL auto_increment,
>   `pmid` int(11) default NULL,
>   `url` longtext,
>   `isComplete` tinyint(1) default '1',
>   `title` longtext,
>   `searchdescription` longtext,
>   `affiliation` varchar(255) default NULL,
>   `journal` varchar(255) default NULL,
>   `author` varchar(255) default NULL,
>   `date` varchar(255) default NULL,
>   `expectedMotifCount` int(11) default '1',
>   `tracking_status` varchar(255) default NULL,
>   `comment` longtext,
>   `score` float default NULL,
>   PRIMARY KEY  (`id`),
>   KEY `pmid` (`pmid`),
>   KEY `pmid_2` (`pmid`),
>   KEY `journal_a` (`journal`),
>   KEY `journal_d` (`journal`),
>   KEY `date_a` (`date`),
>   KEY `date_d` (`date`),
>   KEY `trackingStatus_a` (`tracking_status`),
>   KEY `trackingStatus_d` (`tracking_status`),
>   KEY `pmid_a` (`pmid`),
>   KEY `pmid_d` (`pmid`),
>   KEY `id_a` (`id`)
> ) ENGINE=InnoDB AUTO_INCREMENT=172676 DEFAULT CHARSET=latin1;
> ``` ````

---

|  |  |
| --- | --- |
| ``` This file was generated with SQL Manager 2005 for MySQL (www.mysqlmanager.com) at 4/24/2009 1:22 PM ``` |  |
