# Supplementary material for: A proposed syntax for Minimotif Semantics, version 1
Source: BMC Genomics. 2009 Aug 5;10:360. doi: 10.1186/1471-2164-10-360 (PMC2733157; doi:10.1186/1471-2164-10-360)
Supplement: Additional file 2 — Database Documentation files. File of documentation of the MySQL data model. [file 1471-2164-10-360-S2.zip › documentation/Tables/reviewevent.html]

reviewevent


|  |  |
| --- | --- |
| ``` 155.37.104.15/expertsystem - expertsystem on 155.37.104.15 ``` |  |

reviewevent

Descriptions

InnoDB free: 31744 kB

Fields

**PK**  **Name**  **Data type**  **Size**  **Precision**  **Values**  **Default**  **Auto Increment**  **Binary**  **Not null**  **Unsigned**  **Zero Fill**  **Unique** |  | id | INTEGER | 10 | 0 |  |  |  |  |  |  |  |  | |  | complete | INTEGER | 1 | 0 |  | 0 |  |  |  |  |  |  | |  | entry | TEXT | 0 | 0 |  |  |  |  |  |  |  |  | |  | comment | TEXT | 0 | 0 |  |  |  |  |  |  |  |  | |  | time | TIMESTAMP | 0 | 0 |  | CURRENT\_TIMESTAMP |  |  |  |  |  |  | |  | test | TINYINT | 1 | 0 |  |  |  |  |  |  |  |  | |  | status | VARCHAR | 255 | 0 |  |  |  |  |  |  |  |  | | | | | | | | | | | | | |

Indices

**Name**  **Fields**  **Unique**  **Collation**  **Full Text** | PRIMARY | id |  | Ascending |  | | | | | |

Foreign Keys

There are no foreign keys for table reviewevent

Triggers

There are no triggers for table reviewevent

Options

**TransactSafe**  **TableType**  **Row Format**  **Check Sum**  **Delay Key Write**  **Pack Keys**  **Temporary**  **Min Rows**  **Max Rows**  **Union** |  | InnoDB | Ascending |  |  |  |  | 0 | 0 |  | | | | | | | | | | |

Definition

> ```` ```
> CREATE TABLE `reviewevent` (
>   `id` int(10) unsigned NOT NULL auto_increment,
>   `complete` int(1) unsigned NOT NULL default '0',
>   `entry` text,
>   `comment` text,
>   `time` timestamp NOT NULL default CURRENT_TIMESTAMP on update CURRENT_TIMESTAMP,
>   `test` tinyint(1) default NULL,
>   `status` varchar(255) NOT NULL,
>   PRIMARY KEY  (`id`)
> ) ENGINE=InnoDB AUTO_INCREMENT=70583 DEFAULT CHARSET=latin1;
> ``` ````

---

|  |  |
| --- | --- |
| ``` This file was generated with SQL Manager 2005 for MySQL (www.mysqlmanager.com) at 4/24/2009 1:22 PM ``` |  |
