# Supplementary material for: A proposed syntax for Minimotif Semantics, version 1
Source: BMC Genomics. 2009 Aug 5;10:360. doi: 10.1186/1471-2164-10-360 (PMC2733157; doi:10.1186/1471-2164-10-360)
Supplement: Additional file 2 — Database Documentation files. File of documentation of the MySQL data model. [file 1471-2164-10-360-S2.zip › documentation/Triggers/Index.html]

Triggers


|  |  |
| --- | --- |
| ``` 155.37.104.15/expertsystem - expertsystem on 155.37.104.15 ``` |  |

Triggers

**Trigger**  **Table**  **Type**  **Event** | UPDATE\_PMS\_STATUS | pubmedsource\_reviewevent | AFTER | INSERT | | | | |

---

|  |  |
| --- | --- |
| ``` This file was generated with SQL Manager 2005 for MySQL (www.mysqlmanager.com) at 4/24/2009 1:22 PM ``` |  |
