# Supplementary material for: A proposed syntax for Minimotif Semantics, version 1
Source: BMC Genomics. 2009 Aug 5;10:360. doi: 10.1186/1471-2164-10-360 (PMC2733157; doi:10.1186/1471-2164-10-360)
Supplement: Additional file 2 — Database Documentation files. File of documentation of the MySQL data model. [file 1471-2164-10-360-S2.zip › documentation/Views/comparimotif_sh3_relevance.html]

comparimotif\_sh3\_relevance


|  |  |
| --- | --- |
| ``` 155.37.104.15/expertsystem - expertsystem on 155.37.104.15 ``` |  |

comparimotif\_sh3\_relevance

Descriptions

There is no description for view comparimotif\_sh3\_relevance

Columns

**Column**  **Type** | consensus | VARCHAR | | relevance | DECIMAL | | |

Definition

> ```` ```
> CREATE ALGORITHM=UNDEFINED DEFINER=`root`@`localhost` SQL SECURITY DEFINER VIEW `comparimotif_sh3_relevance` AS 
>   select 
>     `comparimotif_sh3`.`consensus` AS `consensus`,
>     (count(0) / 366) AS `relevance` 
>   from 
>     `comparimotif_sh3` 
>   where 
>     (`comparimotif_sh3`.`score` > 1) 
>   group by 
>     `comparimotif_sh3`.`consensus` 
>   order by 
>     count(0);
> ``` ````

---

|  |  |
| --- | --- |
| ``` This file was generated with SQL Manager 2005 for MySQL (www.mysqlmanager.com) at 4/24/2009 1:22 PM ``` |  |
