# Supplementary material for: A proposed syntax for Minimotif Semantics, version 1
Source: BMC Genomics. 2009 Aug 5;10:360. doi: 10.1186/1471-2164-10-360 (PMC2733157; doi:10.1186/1471-2164-10-360)
Supplement: Additional file 2 — Database Documentation files. File of documentation of the MySQL data model. [file 1471-2164-10-360-S2.zip › documentation/Views/motif_modifications.html]

motif\_modifications


|  |  |
| --- | --- |
| ``` 155.37.104.15/expertsystem - expertsystem on 155.37.104.15 ``` |  |

motif\_modifications

Descriptions

There is no description for view motif\_modifications

Columns

**Column**  **Type** | modification | VARCHAR | | count(\*) | BIGINT | | |

Definition

> ```` ```
> CREATE ALGORITHM=UNDEFINED DEFINER=`root`@`155.37.104.250` SQL SECURITY DEFINER VIEW `motif_modifications` AS 
>   select 
>     distinct `motif`.`modification` AS `modification`,
>     count(0) AS `count(*)` 
>   from 
>     `motif` 
>   group by 
>     `motif`.`modification`;
> ``` ````

---

|  |  |
| --- | --- |
| ``` This file was generated with SQL Manager 2005 for MySQL (www.mysqlmanager.com) at 4/24/2009 1:22 PM ``` |  |
