# Supplementary material for: A proposed syntax for Minimotif Semantics, version 1
Source: BMC Genomics. 2009 Aug 5;10:360. doi: 10.1186/1471-2164-10-360 (PMC2733157; doi:10.1186/1471-2164-10-360)
Supplement: Additional file 2 — Database Documentation files. File of documentation of the MySQL data model. [file 1471-2164-10-360-S2.zip › documentation/Views/sh3_binders.html]

sh3\_binders


|  |  |
| --- | --- |
| ``` 155.37.104.15/expertsystem - expertsystem on 155.37.104.15 ``` |  |

sh3\_binders

Descriptions

There is no description for view sh3\_binders

Columns

**Column**  **Type** | sequence | VARCHAR | | mtype | VARCHAR | | |

Definition

> ```` ```
> CREATE ALGORITHM=UNDEFINED DEFINER=`root`@`localhost` SQL SECURITY DEFINER VIEW `sh3_binders` AS 
>   select 
>     `motif`.`sequence` AS `sequence`,
>     `motif`.`mtype` AS `mtype` 
>   from 
>     (((`motif` join `motif_source`) join `ref_molecule`) join `ref_domain`) 
>   where 
>     ((`motif_source`.`target` = `ref_molecule`.`id`) and (`ref_domain`.`domain` = _latin1'SH3') and (`ref_domain`.`id` = `ref_molecule`.`ref_domain`) and (`motif`.`id` = `motif_source`.`motif`));
> ``` ````

---

|  |  |
| --- | --- |
| ``` This file was generated with SQL Manager 2005 for MySQL (www.mysqlmanager.com) at 4/24/2009 1:22 PM ``` |  |
