# Supplementary material for: A proposed syntax for Minimotif Semantics, version 1
Source: BMC Genomics. 2009 Aug 5;10:360. doi: 10.1186/1471-2164-10-360 (PMC2733157; doi:10.1186/1471-2164-10-360)
Supplement: Additional file 2 — Database Documentation files. File of documentation of the MySQL data model. [file 1471-2164-10-360-S2.zip › documentation/Views/sh3_binders_t.html]

sh3\_binders\_t


|  |  |
| --- | --- |
| ``` 155.37.104.15/expertsystem - expertsystem on 155.37.104.15 ``` |  |

sh3\_binders\_t

Descriptions

There is no description for view sh3\_binders\_t

Columns

**Column**  **Type** | sequence | VARCHAR | | tally | BIGINT | | |

Definition

> ```` ```
> CREATE ALGORITHM=UNDEFINED DEFINER=`root`@`localhost` SQL SECURITY DEFINER VIEW `sh3_binders_t` AS 
>   select 
>     `m`.`sequence` AS `sequence`,
>     (
>   select 
>     count(0) AS `count(*)` 
>   from 
>     `sh3_binders` 
>   where 
>     ((`sh3_binders`.`sequence` regexp `m`.`sequence`) and (`sh3_binders`.`mtype` like _latin1'%Lex%'))) AS `tally` 
>   from 
>     `sh3_binders` `m` 
>   where 
>     (`m`.`mtype` like _latin1'%Conce%') 
>   group by 
>     `m`.`sequence`;
> ``` ````

---

|  |  |
| --- | --- |
| ``` This file was generated with SQL Manager 2005 for MySQL (www.mysqlmanager.com) at 4/24/2009 1:22 PM ``` |  |
