# Supplementary material for: A proposed syntax for Minimotif Semantics, version 1
Source: BMC Genomics. 2009 Aug 5;10:360. doi: 10.1186/1471-2164-10-360 (PMC2733157; doi:10.1186/1471-2164-10-360)
Supplement: Additional file 2 — Database Documentation files. File of documentation of the MySQL data model. [file 1471-2164-10-360-S2.zip › documentation/Views/test_proteolytic_motifs.html]

test\_proteolytic\_motifs


|  |  |
| --- | --- |
| ``` 155.37.104.15/expertsystem - expertsystem on 155.37.104.15 ``` |  |

test\_proteolytic\_motifs

Descriptions

There is no description for view test\_proteolytic\_motifs

Columns

**Column**  **Type** | sequence | VARCHAR | | description | VARCHAR | | |

Definition

> ```` ```
> CREATE ALGORITHM=UNDEFINED DEFINER=`root`@`155.37.104.250` SQL SECURITY DEFINER VIEW `test_proteolytic_motifs` AS 
>   select 
>     `motif`.`sequence` AS `sequence`,
>     `ref_knownactivity`.`description` AS `description` 
>   from 
>     ((`motif` join `motif_source`) join `ref_knownactivity`) 
>   where 
>     ((`ref_knownactivity`.`activitySubclass` like _latin1'%proteolyz%') and (`motif`.`id` = `motif_source`.`motif`) and (`ref_knownactivity`.`id` = `motif_source`.`knownActivity`));
> ``` ````

---

|  |  |
| --- | --- |
| ``` This file was generated with SQL Manager 2005 for MySQL (www.mysqlmanager.com) at 4/24/2009 1:22 PM ``` |  |
